# Identification of novel marker-trait associations for agronomic traits in bread wheat under WANA environments through GWAS

**DOI:** 10.1371/journal.pone.0329681

**Published:** 2025-08-08

**Authors:** Fatima Henkrar, Zakaria El Gataa, Khaoula Lahrichi, Wuletaw Tadesse

**Affiliations:** 1 Laboratory of Plant Biotechnology and Physiology, Center of Plant and Microbial Biotechnology, Biodiversity and Environment, Faculty of Sciences, Mohammed V University in Rabat, Rabat, Morocco; 2 The International Center for Agricultural Research in the Dry Areas (ICARDA), Rabat, Morocco; Amity University, INDIA

## Abstract

Bread wheat (*Triticum aestivum* L.) is a primary staple crop globally and holds particular significance in the West Asia and North Africa region, where it plays a central role in food security and dietary intake. However, rising temperatures, decreased precipitation, and other climate-related stresses increasingly threaten local production. Understanding the genetic basis of agronomic traits is essential for developing high-yielding wheat varieties under harsh conditions. In this study, 191 spring bread wheat genotypes developed by ICARDA for drought and heat tolerance were evaluated across seven environments in Morocco, Sudan, Egypt, and Lebanon for five key agronomic traits. A genome-wide association study using 12,954 SNPs identified 37 significant marker-trait associations (MTAs). The highest number of MTAs was detected for plant height (19), followed by days to maturity (14), thousand kernel weight (5) and three each for days to heading and grain yield. Among these, five MTAs were associated with multiple traits, particularly days to heading, days to maturity and plant height. These included tplb0057m23_716 (5A), AX-94506854 (1B), RAC875_c17628_867 (2A), and wsnp_Ra_rep_c69692_67234463 (3A), validated by both BLINK and FarmCPU models. Candidate genes associated to key traits were identified, such as *TraesCS5A02G261900* (tplb0057m23_716), regulating flowering time, pollen maturation and plant height, and *TraesCS2D02G583000* (tplb0049b24_1152), associated to grain yield through its role in grain peroxidase activity. Other notable genes include *TraesCS5B02G022300* (AX-94476767), encoding N-carbamoyl putrescine amidohydrolase (*TaNPLP1−1*), involved in abiotic stress tolerance, and *TraesCS7D02G102000* (wsnp_Ex_c2054_3852564), associated with cuticular wax biosynthesis for drought and heat protection. Further validation of these SNPs, using inbred lines tested across diverse environments, is required. Once validated, these SNP-derived KASP markers will support breeding for heat- and drought-tolerant lines with desirable agronomic traits.

## Introduction

Wheat (*Triticum aestivum* L.) is the most widely cultivated cereal crop globally and serves as a primary source of calories and protein for over one-third of the world’s population. In West Asia and North Africa (WANA) region, wheat holds particular importance as a staple food, contributing significantly to daily caloric intake. In several WANA countries, including Morocco, Algeria, and Tunisia, per capita wheat consumption ranges from 179 to 196 kg annually, among the highest globally [1,2]. Despite its dietary significance, the WANA region struggles to meet domestic wheat demand, with over half of its consumption reliant on imports. These constraints are largely driven by frequent droughts, land degradation, and more recently, the intensifying impacts of climate change. Projections indicate a 2–3 °C rise in mean temperature and a 10–20% decline in rainfall by 2050 across the WANA region [3], conditions that are expected to intensify the frequency of heatwaves and drought. Drought and heat stress negatively affect wheat productivity by impairing critical physiological and developmental processes. These stresses can lead to shortened grain-filling periods, reduced photosynthetic efficiency, disrupted pollen viability, stunted growth, and diminished root development, all of which collectively reduce grain yield and key agronomic performance traits [4]. As a result, enhancing stress resilience in wheat is essential for sustaining productivity in WANA environments. To effectively mitigate the detrimental impacts of drought and heat stress on wheat productivity, it is imperative to explore the genetic basis of stress tolerance.

Genome-wide association studies (GWAS) provide a robust framework for dissecting the genetic architecture of complex traits associated with stress adaptation. GWAS enables the identification of single nucleotide polymorphisms (SNPs) and candidate genes associated with traits such as flowering time, plant height, grain weight, and stress tolerance. These genes serve as valuable targets for molecular breeding and biotechnological strategies to develop drought-tolerant wheat varieties [5]. In parallel, integrating GWAS with climate modeling facilitates the prediction of genotype performance under future climatic scenarios, thereby supporting targeted variety development. This is exemplified by initiatives such as the Heat and Drought Wheat Improvement Consortium (HeDWIC), which seeks to harness advanced genomics to enhance wheat resilience under abiotic stress [6–8]. Numerous GWAS have identified Quantitative Trait loci (QTLs) associated with key agronomic traits in wheat under heat and drought stress. For example, Liu et al. [9] identified QTLs such as *Qgns.cas-3A.2* on chromosome 3A, linked to yield stability under drought, while Fu et al. [10] identified 15 stable QTLs (*QHST1–QHST15*) potentially associated with heat stress tolerance in wheat at the seedling stage. Touzy et al. [11] found QTLs (*QTL.01-QTL.10*) related to post-anthesis heat stress and senescence, and Bashir et al. [12] identified two quantitative trait nucleotides *Qql.iari-2A.1_Fe* (AX-94461119) and *Qql.iari-7D_ Zn* (AX-95220192) for grain iron and zinc content under heat stress, validated using KASP markers. A meta-QTL analysis by Kumar et al. [13] consolidated 85 stable and robust MQTLs associated with thermotolerance in wheat. Among these, seven promising MQTLs, designated as breeders’ MQTLs (*MQTL2A.4*, *MQTL3B.2*, *MQTL5B.3*, *MQTL6D.1*, *MQTL7A.1*, *MQTL7B.1*, and *MQTL7B.3*), were recommended for use in marker-assisted breeding to improve heat stress tolerance in wheat. In addition, Tahmasebi et al. [14] identified a major QTL (7Dacc/cat10) associated with terminal drought and heat escape, linked to increased thousand kernel weight and stable yield without significant genotype-by-environment interaction. Building on these findings, several candidate genes have been proposed to explain the observed phenotypic variation. For instance, *GA20ox* and *CaaX prenyl protease 2* were associated to grain yield stability and canopy temperature under combined heat–drought stress [15]. A SNP (100035706) was associated with the *DMAS1-A* gene, involved in iron acquisition [16]. Additionally, *TRAESCS1D02G029500* (*CSC1*) and *TRAESCS5B02G302500* (*GME*) were identified as drought-responsive genes [17], and glycosyl hydrolase genes 17 were associated with yield stability under drought stress [9].

Expending upon earlier GWAS studies, which were often limited to single environments, this study aims to investigate the genetic architecture of key agronomic traits across diverse environments in the WANA region. Specifically, we aim to identify genomic regions and SNPs associated with days to heading, days to maturity, plant height, thousand kernel weight, and grain yield across a genetically diverse panel of spring bread wheat genotypes evaluated in multiple environments. We hypothesize that multi-environment GWAS will reveal both stable and environment-specific loci, thereby improving understanding of genotype-by-environment interactions and identifying candidate genes underlying stress adaptation.

## Materials and methods

### Plant materiel and field experiment

**Fig 1:**
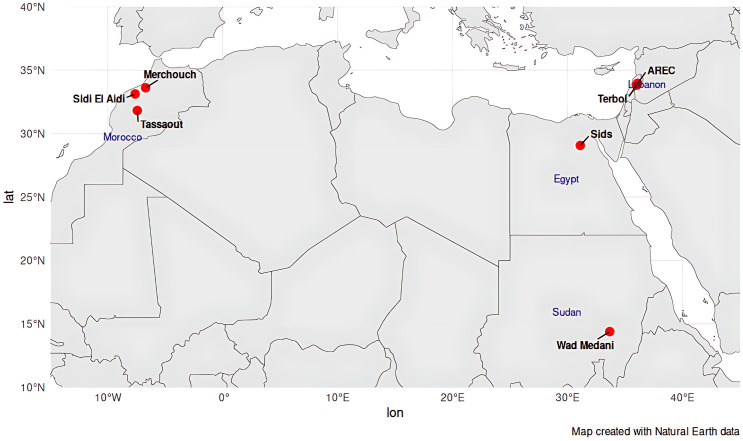
Geographic locations of the seven experimental stations used in the study. This map was generated in R using public domain data from Natural Earth (http://www.naturalearthdata.com/) and is intended for illustrative purposes only.

The GWAS panel included 191 spring bread wheat genotypes developed at ICARDA (S1 Table). These advanced lines were selected for their potential in evaluating drought and heat tolerance as well as yield performance and were characterized by significant phenotypic variation. The field experiment was conducted across seven experimental stations (Fig 1), each representing distinct agro-climatic conditions. These stations were: the ICARDA Research Station in Merchouch (33°36’29.34”N, 6°42’55.19”W); National Institute of Agronomic Research (INRA) Station in Sidi El Aidi (33°7’27.50”N, 7°37’30.15”W); and INRA Station in Tassaout (31°49’15.51”N, 7°26’15.71”W) in Morocco; the Gezira Research Station (14°23’34.31”N, 33°39’34.32”E) in Wad Medani, Sudan; Sids Station at the Agricultural Research Center (ARC) in Beni Suef, Egypt (29°3′58.06″N, 31°5′57.79″E); and the ICARDA Terbol Station (33°48’43.29”N, 35°59’26.36”E) and the Advancing Research Enabling Communities Center (AREC)-AUB Farm in Beirut, Lebanon (33°57’30.29”N, 36°4’41.42”E).

Merchouch and Sidi El Aidi were characterized by drought conditions, while Tassaout, Terbol, and AREC were grown under irrigation conditions. Wad Medani, in contrast, is known for its high temperatures, providing a heat-stress environment. Detailed information on temperature ranges, total rainfall, soil type, and pH is available in Ed-Daoudy et al. [18], except for Wad Madani, where the soil is classified as vertisol with a pH of 8.5, an annual temperature range of 27.66ºC to 39.78ºC, and an average annual precipitation of 306.4 mm. At the Sids station, the soil is loamy clay with a pH ranging from 7.7 to 8.0, the annual temperature range is between 17.73ºC and 29.78ºC, and the average annual precipitation is between 7 and 20 mm.

The GWAS panel was evaluated using a row–column design with replicated controls to account for spatial variability across the field. Each genotype was sown in a 3-meter-long plot consisting of six rows, with inter-row spacing of 20 cm. The experiments were conducted during two consecutive growing seasons (2017 and 2018).

### Phenotypic trait evaluation and data analysis

All genotypes in the GWAS panel were phenotyped for five quantitative traits: days to heading (DHE), days to maturity (DMA), plant height (PLH), thousand kernel weight (TKW), and gain yield (GY) at Merchouch, Terbol, Tassaout (except TKW), AREC (except TKW), and Sids (except TKW). However, only GY was recorded at Sidi El Aidi and Wad Madani.

PLH was measured at physiological maturity by averaging the heights of three randomly selected plants from the central rows of each plot, avoiding border plants. The measurement was taken from the soil surface to the tip of the spike, excluding the awns. DHE were recorded as the number of days from seeding until 50% of the spikes had fully emerged from the leaf sheath. DMA were recorded as the number of days from seeding until 50% of the spikes had completely turned yellow. For TKW, grain samples were counted and weighed only at the Merchouch (Morocco) and Terbol (Lebanon) sites, where electronic seed counters were available. The average weight of 1,000 grains was then calculated for each sample. GY was recorded in kilograms per plot after harvesting the entire plot, and then converted to ton nes per hectare.

Phenotypic data were analyzed using R packages. The mean, standard deviation, and coefficient of variation (CV) were calculated using the RcmdrMisc package [19]. Broad-sense heritability within a single environment (*H²*_*within*_) was estimated for each trait under the assumption of no replication, using the formula [20]:


$$H_{with\in^{2}=\frac{\sigma_{P}^{2}}{\sigma_{P}^{2}+\sigma_{e}^{2}}}$$

Where $\sigma_{P}^{2}$ is the phenotypic variance and $\sigma_{P}^{2}$ is the assumed residual (error) variance. Due to the absence of plot-level replication, phenotypic variance was calculated as the variance of observed trait values across genotypes, and a fixed residual variance ($\sigma_{e}^{2}$=10) was assumed based on prior field trial estimates. Additionally, *H²*_*across*_ was estimated across environments using a linear mixed model fitted with the lme4 package in R and was computed using the variance components estimated from the model as follows [20]:


$$H_{across}^{2}=\frac{\sigma_{G}^{2}}{\sigma_{G}^{2}+\frac{\sigma_{GxE}^{2}}{n_{E}}+\frac{\sigma_{\varepsilon }^{2}}{n_{E}\cdot n_{R}}}$$

Where, $\sigma_{G}^{2}$ is the genotypic variance,$\sigma_{GxE}^{2}$ is the genotype-by-environment interaction variance, $\sigma_{\varepsilon }^{2}$ is the residual variance, $n_{E}$ is the number of environments, and $n_{R}$ is the average number of replications per genotype per environment.

The least significant difference (LSD) was determined from the combined analysis using the LSD.test function in the agricolae package [21]. Additionally, best linear unbiased estimations (BLUEs) across all environments were calculated using TASSEL v5 software with the following model: y = μ + genotype + environment + e.

Principal component analysis (PCA) was performed using the FactoMineR [22] and factoextra [23] packages. The correlation matrix was generated using BLUEs and visualized using the PerformanceAnalytics package [24].

### Genotyping

A total of 191 bread wheat genotypes were analyzed using the Wheat Illumina iSelect 15K SNP array. For DNA extraction, flag leaves were collected from three randomly selected plants per genotype at the appropriate growth stage. The leaf samples were bulked, placed in well plates, and sent to the SGS Institut Fresenius GmbH, TraitGenetics Section, in Gatersleben, Germany (https://sgs-institut-fresenius.de/gesundheit-und-ernaehrung/traitgenetics), for DNA isolation and genotyping. After data collection, quality control and SNP filtering were performed using TASSEL v5 software. Monomorphic markers and SNPs with more than 10% missing data were removed. SNPs with a minor allele frequency (MAF) below 5% and those with heterozygosity greater than 10% were also excluded to minimize the influence of rare alleles and genotyping errors. After filtering, 12,954 high-quality SNPs were retained for GWAS analysis.

### Population structure and linkage disequilibrium

Population structure was assessed using a Bayesian model-based approach implemented in STRUCTURE software, v2.3.4 [25]. The number of subpopulations (k) was tested in the range of K = 2–10, using a burn-in period of 20,000 iterations followed by 50,000 iterations of the Monte Carlo Markov Chain (MCMC). The optimal number of subpopulations was determined using the ∆K method, calculated with the pophelper R package [26].

Principal component analysis (PCA) was performed on the filtred set of polymorphic SNPs using the GAPIT R package v3 [27] and confirmed using the prcomp function. Kinship relationships were estimated in TASSEL and visualized using the heatmap.2 function from the gplots package [28]. Linkage disequilibrium (LD) was evaluated in TASSEL. Pairwise LD (r^2^) between SNP markers was calculated, and both chromosome-wise and genome-wide LD decay were analyzed. The LD decay curve was visualized by plotting r^2^ values against physical distance (in base pairs), following the method described by Remington et al. [29].

### Genome-wide association analysis and gene annotation

Genome-wide association analysis was conducted using 12,954 SNP markers, employing both the FarmCPU [30] and BLINK [31] models implemented in the GAPIT R package v3 [27]. To control for false positives, false discovery rate (FDR) correction was applied to all MTAs using the Benjamini and Hochberg method [32], and markers with corrected *p*-values < 0.05 were considered statistically significant. Manhattan plots were generated using CMplot v3.4.0 [33]. A Bonferroni-adjusted threshold was applied to determine significance (*p* = 0.05/12,954), corresponding to -log_10_(*p*) > 5.41. SNPs exceeding this threshold were considered significant and visualized in the Manhattan plots. Candidate gene mining was done using the Ensembl wheat server (http://plants.ensembl.org/Triticum_aestivum/Info/Index). Functional analysis of the resulting candidate genes, including their molecular function and biological processes, was conducted using the UniProt database (https://www.uniprot.org).

## Results

### Phenotypic variation and heritability

The agronomic traits evaluated across multiple environments (Table 1) exhibited significant variation in means, variability and broad-sense heritability. The Merchouch environment recorded the highest heritability for DHE and DMA (*H²*_*within*_ = 0.88 and 0.96, respectively), reflecting strong genetic control despite environmental variability. GY exhibited considerable variability, ranging from a high of 8.37 t/ha at Terbol to a low of 2.84 t/ha at Wad Madani. However, GY was characterized by high coefficients of variation (CV ranging from 10.02% to 23.06%) and consistently low heritability, particularly at Sidi El Aidi (*H²*_*within*_ = 0.03) and Wad Madani (*H²*_*within*_ = 0.07), highlighting the dominant role of environmental factors in influencing yield. PLH demonstrated high heritability across all environments (*H²*_*within*_ ranging from 0.73–0.87), indicating a stable genetic contribution. TKW, evaluated at Merchouch and Terbol, also showed high heritability (*H²*_*within*_ = 0.72–0.90), with greater phenotypic variability at Merchouch (CV = 14.35%).

**Table 1:** Descriptive statistics and genetic parameters for agronomic traits across environments.

Environment	Trait	Mean	Max	Min	SD	CV (%)	*H²*_*within*_
AREC	DHE	122.33	129.00	116.00	2.79	2.28	0.44
	DMA	162.94	169.00	154.00	2.99	1.83	0.47
	GY	7.82	11.70	3.51	1.44	18.37	0.17
	PLH	94.29	110.00	60.00	7.76	8.23	0.86
Merchouch	DHE	104.92	120.00	41.00	8.69	8.28	0.88
	DMA	155.64	185.00	77.00	16.54	10.63	0.96
	GY	5.46	8.50	3.40	1.02	18.74	0.30
	PLH	98.87	121.00	70.00	8.33	8.43	0.87
	TKW	30.12	38.74	21.11	4.32	14.35	0.90
Sidi El Aidi	GY	5.98	7.62	4.43	0.60	10.02	0.03
Sids	DHE	103.75	108.00	101.00	1.47	1.42	0.18
	DMA	155.40	162.00	150.00	2.95	1.90	0.47
	GY	8.05	12.03	4.63	1.30	16.14	0.14
	PLH	114.12	130.00	90.00	7.79	6.83	0.86
Tassaout	DHE	95.88	112.00	83.00	3.78	3.94	0.59
	DMA	143.36	149.00	138.00	2.80	1.96	0.44
	GY	6.11	9.79	0.68	1.41	23.06	0.17
	PLH	89.29	100.00	80.00	5.22	5.85	0.73
Terbol	DHE	145.93	153.00	141.00	2.82	1.93	0.44
	DMA	187.97	191.00	184.00	1.63	0.86	0.21
	GY	8.37	12.29	1.25	1.58	18.85	0.20
	PLH	100.52	120.00	80.00	5.80	5.77	0.77
	TKW	43.84	58.00	27.06	5.09	11.61	0.72
Wad Madani	GY	2.84	3.96	2.11	0.44	15.37	0.07

DHE, days to heading; DMA, days to maturity; GY, grain yield; PLH, plant height; TKW, thousand kernel weight; SD, standard deviation; CV, coefficient of variation; *H²*_*within*_, broad-sense heritability.

Table 2 compares broad-sense heritability across and within environments for each trait. Notably, GY shows the lowest across-environment heritability (*H²*_*across*_ = 0.263), consistent with its high environmental sensitivity, whereas TKW and PLH display relatively stable genetic control (*H²*_*across*_ = 0.761 and 0.610, respectively). In general, within-environment heritability estimates (*H*^*2*^_*within*_) were higher than across-environments estimates (*H*^*2*^_*across*_), indicating that environmental context plays a significant role in trait expression, and that the genetic control of the trait appears stronger when assessed under stable and uniform conditions.

**Table 2:** Comparison of broad-sense heritability estimates across and within environments for agronomic traits.

Trait	*H²*_*across*_	Range of *H²*_*within*_
GY	0.263	0.03–0.30
TKW	0.761	0.72–0.90
PLH	0.61	0.73–0.87
DHE	0.414	0.18–0.88
DMA	0.297	0.21–0.96

GY, grain yield; TKW, thousand kernel weight; PLH, plant height; DHE, days to heading; DMA, days to maturity; *H²*_*across*_, broad-sense heritability estimated across environments; *H²*_*within*_, broad-sense heritability estimated within individual environments.

### Genotypic performance across environments

Table 3 presents the top 20 high-yielding genotypes across seven environments. Grain yield varied substantially across locations, with some genotypes excelling in specific environments. For instance, G19 (ASEEL-1//MILAN/PASTOR/3/SHAMISS-3) achieved the highest yield (12.03 t/ha) at Sids, while G171 (TEMPORALERA M 87*2/KONK//FAYEQ-1) and G80 (GEMMEIZA-10/SHAMISS-3) recorded notable performances at Terbol (10.44 t/ha and 10.43 t/ha, respectively). In terms of stability, G101 (JAWAHIR-9/ETBW 4920) and G171 (TEMPORALERA M 87*2/KONK//FAYEQ-1) consistently performed well across four testing locations: Merchouch, Sidi El Aidi, Sids, and Terbol. G46 (CHAM-8/RUTH-3//ZAIN-2) also exhibited strong performance in four environments, including AREC, Merchouch, Tassaout, and Wad Madani, demonstrating potential adaptability in diverse agro-ecological zones. Several other genotypes ranked among the top 20 in two to three environments, highlighting promising candidates for multi-environment cultivation.

**Table 3:** Top 20 high-yielding genotypes across the seven environments (t/ha).

Genotype	GY AREC	Genotype	GY Merchouch	Genotype	GY Sidi El Aidi	Genotype	GY Sids	Genotype	GY Tasaout	Genotype	GY Terbol	Genotype	GY Wad Madani
**G46**	10.175	**G101**	7.179	**G101**	7.441	**G101**	9.844	**G46**	9.04	**G101**	9.764	**G46**	4.727
**G22**	9.254	**G171**	6.649	**G171**	7.622	**G171**	9.844	**G127**	8.692	**G171**	10.436	**G127**	3.992
**G28**	9.094	**G46**	6.450	**G57**	6.830	**G22**	10.094	**G22**	8.252	**G80**	10.430	**G45**	4.234
**G45**	9.518	**G127**	6.612	**G115**	7.185	**G57**	9.938	**G28**	8.392	**G136**	9.679	**G112**	4.188
**G10**	9.152	**G28**	7.810	**G148**	6.896	**G80**	9.969	**G45**	8.080	**G156**	9.915	**G47**	4.227
**G136**	9.649	**G57**	7.049	**G167**	7.285	**G10**	9.844	**G80**	7.900	**G172**	9.916	**G56**	4.281
**G168**	9.795	**G168**	6.665	**G172**	6.985	**G148**	9.844	**G112**	9.792	**G40**	9.815	**G63**	4.563
**G42**	9.020	**G181**	6.844	**G181**	6.974	**G156**	9.844	**G115**	8.120	**G110**	9.565	**G67**	4.117
**G47**	9.108	**G21**	6.568	**G72**	7.400	**G56**	9.781	**G167**	7.660	**G135**	10.305	**G77**	4.031
**G68**	9.123	**G4**	6.640	**G77**	6.859	**G63**	10.563	**G21**	7.928	**G137**	10.084	**G124**	3.898
**G72**	9.781	**G40**	6.704	**G103**	7.115	**G67**	10.000	**G4**	7.884	**G139**	9.735	**G13**	4.844
**G9**	9.342	**G55**	6.754	**G106**	6.985	**G73**	9.844	**G42**	7.964	**G146**	9.777	**G155**	3.898
**G123**	9.459	**G73**	6.798	**G122**	7.244	**G9**	9.844	**G55**	9.280	**G150**	10.394	**G183**	5.078
**G128**	9.635	**G15**	7.029	**G132**	7.148	**G108**	9.844	**G68**	7.880	**G16**	9.850	**G190**	4.305
**G164**	9.401	**G29**	6.603	**G147**	6.856	**G11**	9.844	**G126**	8.484	**G170**	11.147	**G24**	4.109
**G26**	9.327	**G33**	6.697	**G159**	7.085	**G118**	9.781	**G18**	7.632	**G177**	9.567	**G61**	4.016
**G31**	9.561	**G34**	6.626	**G185**	6.926	**G12**	9.844	**G38**	9.028	**G178**	9.601	**G7**	4.531
**G75**	9.342	**G5**	6.793	**G188**	7.296	**G19**	12.031	**G53**	9.128	**G59**	9.562	**G84**	4.164
**G78**	9.079	**G50**	6.625	**G86**	7.044	**G89**	9.844	**G62**	8.828	**G69**	9.626	**G87**	4.086
**G83**	9.152	**G51**	6.781	**G93**	7.200	**G95**	9.844	**G76**	7.720	**G90**	9.951	**G96**	4.273

Genotypic performance is shown in tons per hectare (t/ha) for each environment. Genotypes are color-coded based on the number of environments in which they ranked among the top 20: pink indicates four environments, green three, and blue two.

### Environmental effect on trait variation

**Fig 2:**
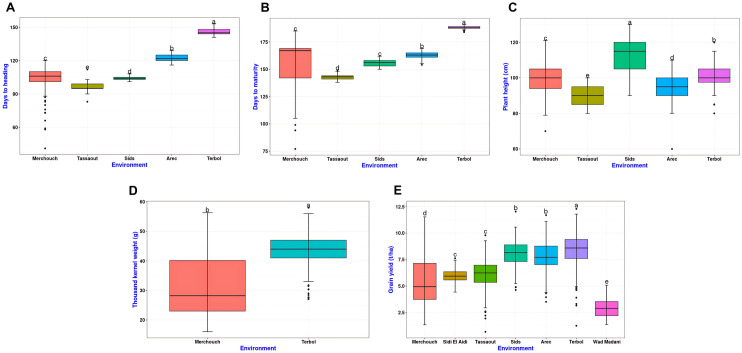
Boxplots summarizing the differences in agronomic traits. **A)** DHE, **B)** DMA, **C)** PLH, **D)** TKW and **E)** GY, across different environments (Merchouch, Sidi EL Aidi, Tassaout, Sids, AREC, Terbol and Wad Madani) for year 2017 and 2018.

The LSD boxplot (Fig 2) illustrates significant differences in DHE, DMA, PLH, TKW, and GY across the seven tested environments. Trait variation was primarily driven by water availability and thermal stress. Environments such as Merchouch and Sidi El Aidi, which experienced drought, exhibited greater variability and lower GY, likely due to water deficits during critical growth stages. In contrast, Tassaout, Terbol, and AREC, which were irrigated, showed higher and more stable grain yields, demonstrating the positive impact of consistent water supply. Among these, Terbol had the highest average yield and the longest heading duration, reflecting a longer and more favorable growing season under irrigation. Conversely, Wad Madani, exposed to high temperatures, recorded the lowest yields, indicating the adverse effects of heat stress on crop productivity.

### Trait correlations and component analysis

**Fig 3:**
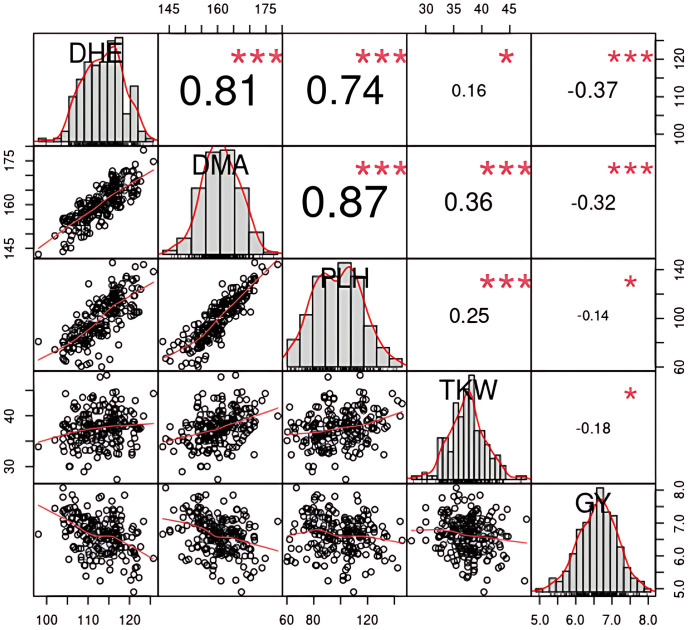
Pearson’s correlation matrix among agronomic traits based on BLUEs. Correlation values (*, **, ***) indicate significance at **p* *< 0.05, 0.01, and 0.001, respectively.

The Pearson correlation matrix (Fig 3) based on BLUE values (S1 Table) reveals strong positive correlations among DHE, DMA, and PLH, with the highest between DHE and DMA (r = 0.87, ***). In contrast, GY displayed negative correlations with these traits and also with TKW, suggesting potential trade-offs between prolonged growth and yield stability under stress. Diagonal histograms in Fig 3 illustrate the approximate normal distributions of most traits, confirming broad phenotypic variation across genotypes. Bivariate scatterplots with fitted lines reinforce the direction and strength of trait associations.

**Fig 4:**
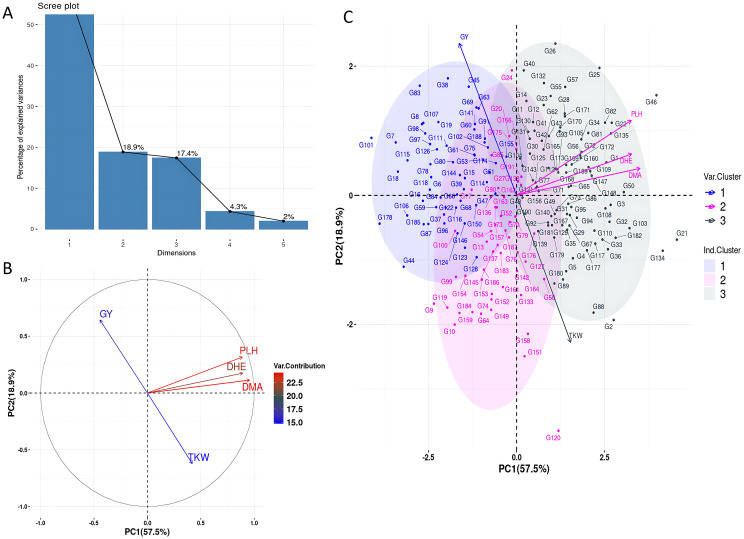
Principal component analysis of agronomic traits and genotypes. **A)** Scree plot of variance explained by components. **B)** Variable-PCA-biplot showing trait contributions. **C)** Genotype-PCA biplot showing genotype distribution based on trait variation.

Principal component analysis was conducted to visualize the relationships among key agronomic traits and wheat genotypes. Fig 4A shows a scree plot of the explained variance ratio: the first principal component (PC1) accounts for 57.5% of the total variance, followed by 18.2% for PC2 and 17.4% for PC3. Together, these three components explain 93.1% of the total variance. In the PCA biplot (Fig 4B and 4C), variables contribute differentially to the principal components. GY is the largest contributor to PC2 (18.9%), capturing trait variation largely independent of other variables. PLH, DHE, and DMA are tightly clustered and contribute strongly to PC1 (57.5%), which reflects the primary trend in trait variation. TKW also contributes notably to PC1 but in the opposite direction to PLH, DHE, DMA, and GY indicating a negative association along this axis. Furthermore, GY and TKW exhibit contrasting loadings on PC1 (−0.444 and 0.420, respectively) and PC2 (0.642 and −0.621), reflecting opposing contributions in two-dimensional space (Table 4). However, both traits contribute similarly to PC3 (0.615 for GY and 0.657 for TKW), a component not represented in the biplot, suggesting that their full relationship is better captured beyond the first two dimensions.

**Table 4:** Coordinates (correlations) of agronomic traits with the first five principal components from PCA.

Trait	PC1	PC2	PC3	PC4	PC5
DHE	0.892	0.175	−0.199	0.361	−0.064
DMA	0.956	0.114	0.062	−0.084	0.248
PLH	0.889	0.317	0.13	−0.251	−0.174
TKW	0.42	−0.621	0.657	0.068	−0.034
GY	−0.444	0.642	0.615	0.106	0.027

### Population structure and linkage disequilibrium

**Fig 5:**
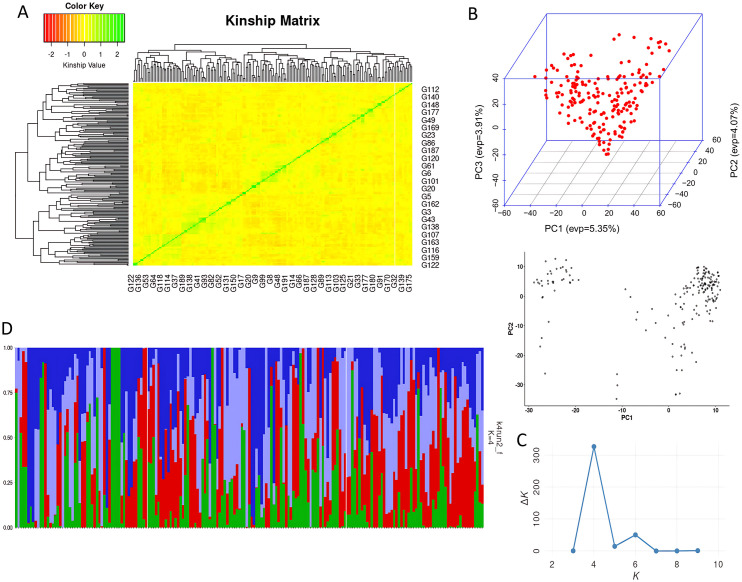
Population structure analysis. **A)** Kinship heatmap. **B)** PCA showing three sub-populations. **C)** ∆K values across K = 2-10 (x-axis). **D)** STRUCTURE plot at K = 4.

The kinship matrix of the 191 wheat genotypes, based on 12,954 SNP markers (Fig 5A), reveals significant genetic relatedness and population structure. Two major clusters are evident, each further divided into sub-clusters. Red indicates low kinship (genetic dissimilarity), while green denotes high kinship (similarity). The PCA plot (Fig 5B) visualizes genetic variation based on the first two PCs, revealing three partially overlapping groups, indicative of continuous variation and admixture. STRUCTURE analysis identified four genetic subpopulations (K = 4) as optimal, supported by a peak ΔK value of 326.69 (Figs 5C and 5D). Subpopulation 1 (red) includes genotypes with West Asian/Mediterranean ancestry (e.g., CHAM, ATTILA) adapted to drought and heat. Subpopulation 2 (green) comprises high-yielding lines adapted to irrigated conditions (e.g., PASTOR, OPATA, and MILAN). Subpopulation 3 (blue) represents admixed background, often from synthetic or three-way crosses. Subpopulation 4 (yellow) contains heat-tolerant lines from CIMMYT’s Heat Tolerant Wheat Screening Nursery (HTWSN), with parents like KAUZ, PBW343, and SOKOLL.

**Fig 6:**
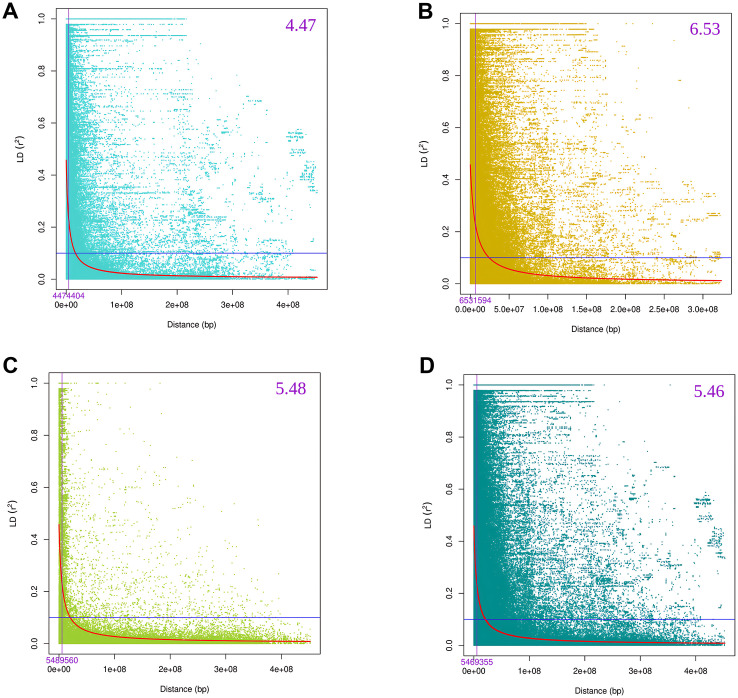
LD decay plots for A, B, D and whole genome. Violet line indicates 50% of maximum LD. Blue line indicates r^2^ threshold at 0.1. X-axis shows LD decay distance (Mb).

Analysis of SNP density across 1 Mb windows (S1 Fig) shows that the B genome has the highest density, especially on chromosomes 2B and 5B, while the D genome has the lowest. Of the 12,954 SNPs used for LD analysis, 4,859 are from genome A, 5,967 from B and 2,064 from D. Pairwise LD analysis (r^2^) reveals that LD decay varies by genome (Fig 6). On average, genome-wide LD dropped below r^2^ = 0.1 at 5.46 Mb. B genome exhibited the longest LD span (6.53 Mb), followed by D (5.48 Mb) and A (4.47 Mb) reflecting differing historical recombination rates and selection pressures.

### Genome-wide association analysis

**Fig 7:**
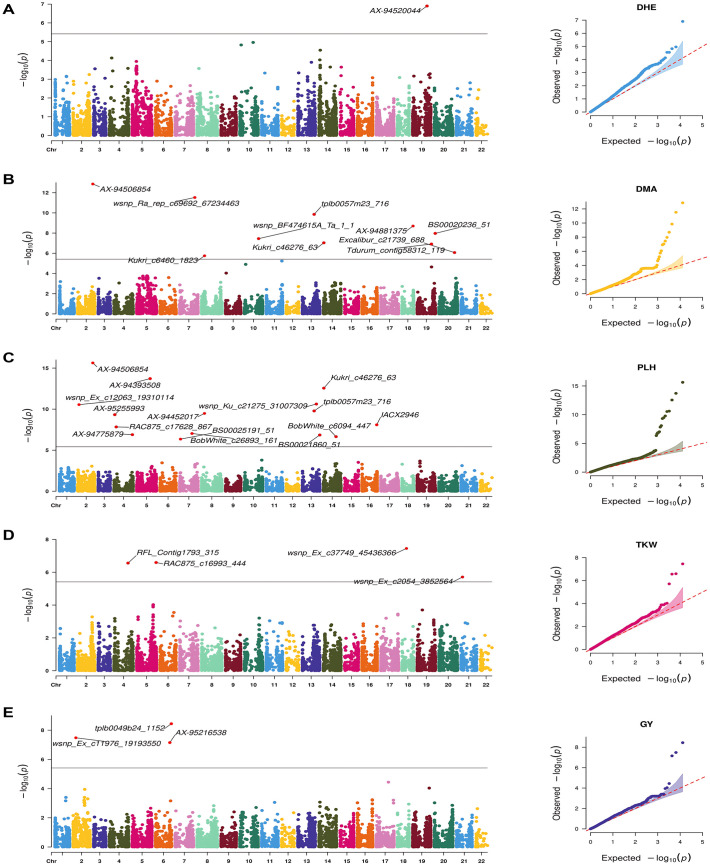
Manhattan and respective-QQ plots for agronomic traits in the 191-wheat association panel using BLINK. **A)** DHE, **B)** DMA, **C)** GY, **D)** PLH and **E)** TKW.

**Fig 8:**
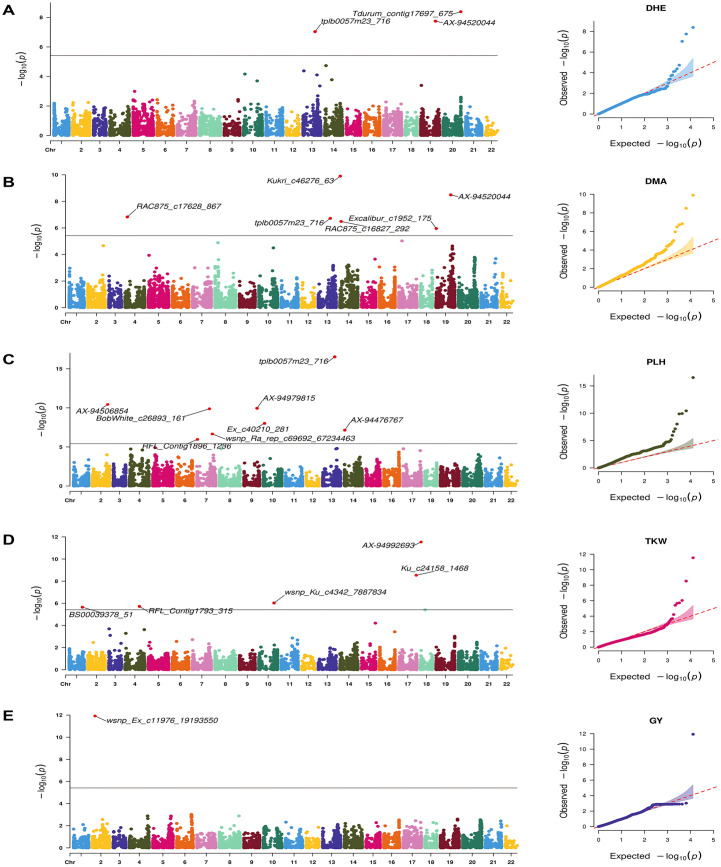
Manhattan and respective-QQ plots for agronomic traits in the 191-wheat association panel using FarmCPU. **A)** DHE, **B)** DMA, **C)** GY, **D)** PLH and **E)** TKW.

The genome-wide association study using 12,954 SNPs identified 37 significant MTAs associated to five key agronomic traits: DHE, DMA, GY, PLH, and TKW. These MTAs were detected across multiple environments using both BLUEs and individual environment datasets (Table 5). Significance was determined using Bonferroni-corrected thresholds and FDR-adjusted *p*-values [32], with all 37 MTAs surpassing the genome-wide significance threshold of 3.85 × 10 ⁻ ⁶ (*p* = 0.05). BLINK and FarmCPU models identified 30 and 16 unique MTAs, respectively, with 9 MTAs shared between both models (Figs 7 and 8).

**Table 5:** MTAs identified in multiple environments (BLUEs and individual environments) for the five agronomic traits in the association panel of spring bread wheat.

Trait	Model	SNP	Chr	Physical position (bp)	Alleles	P.value	MAF	H&B P_Value	Effect	PVE (%)	Environment
DHE	BLINK	**AX-94520044**	7A	579503778	C/T	5.80E-09	0.41	1.07E-05	−0.88	27.11	BLUEs
DHE	FarmCPU	**AX-94520044**	7A	579503778	C/T	1.80E-08	0.41	1.16E-04	−0.89	14.29	BLUEs
DHE	FarmCPU	Tdurum_contig17697_675	7B	699147853	G/A	4.10E-09	0.35	5.32E-05	0.93	8.88	BLUEs
DHE	FarmCPU	**tplb0057m23_716**	5A	475467251	A/G	9.20E-08	0.41	3.97E-04	0.82	10.76	BLUEs
DMA	BLINK	**AX-94506854**	1B	613423513	T/G	1.40E-13	0.05	1.79E-09	−2.67	43.47	BLUEs
DMA	BLINK	AX-94881375	6D	446614420	A/C	2.00E-09	0.50	6.46E-06	0.99	2.43	BLUEs
DMA	BLINK	BS00020236_51	7A	730298189	T/C	1.10E-08	0.18	2.77E-05	1.03	5.65	BLUEs
DMA	BLINK	Excalibur_c21739_688	7B	632491797	A/G	8.50E-07	0.28	1.22E-03	0.85	3.07	BLUEs
DMA	BLINK	**Kukri_c46276_63**	5B	16002366	T/C	9.10E-08	0.33	1.69E-04	−0.90	3.08	BLUEs
DMA	BLINK	Kukri_c6460_1823	3B	117575406	C/A	1.77E-06	0.27	2.29E-03	0.79	2.53	BLUEs
DMA	BLINK	Tdurum_contig58312_119	7A	575268349	T/C	1.21E-07	0.10	1.96E-04	1.27	7.94	BLUEs
DMA	BLINK	**tplb0057m23_716**	5A	475467251	A/G	1.40E-10	0.41	6.03E-07	1.09	3.19	BLUEs
DMA	BLINK	wsnp_BF474615A_Ta_1_1	4A	581869247	T/C	3.50E-08	0.24	7.65E-05	1.19	12.9	BLUEs
DMA	BLINK	**wsnp_Ra_rep_c69692_67234463**	3A	615275885	C/T	3.00E-12	0.40	1.94E-08	−1.19	5.93	BLUEs
DMA	FarmCPU	**AX-94520044**	7A	579503778	C/T	3.20E-09	0.41	2.10E-05	−0.91	15.06	BLUEs
DMA	FarmCPU	Excalibur_c1952_175	7A	6314255	C/T	1.11E-06	0.16	2.40E-03	−1.08	0.22	BLUEs
DMA	FarmCPU	**Kukri_c46276_63**	5B	16002366	T/C	1.30E-10	0.33	1.63E-06	−1.25	0	BLUEs
DMA	FarmCPU	RAC875_c16827_292	5B	50469657	C/T	3.26E-07	0.25	8.44E-04	−1.02	5.81	BLUEs
DMA	FarmCPU	**RAC875_c17628_867**	2A	90764012	G/A	1.49E-07	0.44	6.18E-04	0.91	0	BLUEs
DMA	FarmCPU	**tplb0057m23_716**	5A	475467251	A/G	1.91E-07	0.41	6.18E-04	0.98	6.99	BLUEs
GY	BLINK	AX-95216538	2D	581826458	T/C	7.00E-08	0.27	9.13E-04	−0.19	20.77	BLUEs
GY	BLINK	tplb0049b24_1152	2D	643436189	C/T	3.60E-09	0.22	4.68E-05	−0.61	28.88	Merch
GY	BLINK	**wsnp_Ex_c11976_19193550**	1B	109729520	G/A	3.30E-08	0.22	4.23E-04	0.29	34.53	SEA
GY	FarmCPU	**wsnp_Ex_c11976_19193550**	1B	109729520	G/A	1.20E-12	0.22	1.52E-08	0.33	0	SEA
PLH	BLINK	AX-94393508	2B	542759611	A/G	1.90E-14	0.37	1.25E-10	3.22	6.34	BLUEs
PLH	BLINK	AX-94452017	3B	115396333	T/C	3.30E-10	0.25	6.16E-07	2.78	5.38	BLUEs
PLH	BLINK	**AX-94506854**	1B	613423513	T/G	2.30E-16	0.05	3.04E-12	−6.70	35.31	BLUEs
PLH	BLINK	AX-94775879	2A	747144140	C/T	1.29E-07	0.47	1.40E-04	−1.92	2.46	BLUEs
PLH	BLINK	AX-95255993	2A	31811192	T/C	4.80E-10	0.34	7.70E-07	−2.41	4.11	BLUEs
PLH	BLINK	**BobWhite_c26893_161**	3A	502268559	C/T	6.00E-08	0.49	7.81E-05	2.01	3.04	BLUEs
PLH	BLINK	BobWhite_c6094_447	5B	511697098	T/C	2.25E-07	0.40	2.08E-04	1.91	2.22	BLUEs
PLH	BLINK	BS00021860_51	5A	704973862	T/C	1.41E-07	0.18	1.40E-04	2.21	4.36	BLUEs
PLH	BLINK	BS00025191_51	3A	36474852	T/G	4.47E-07	0.27	3.86E-04	1.72	1.66	BLUEs
PLH	BLINK	IACX2946	6A	599046538	G/A	8.10E-09	0.39	1.17E-05	2.05	2.47	BLUEs
PLH	BLINK	**Kukri_c46276_63**	5B	16002366	T/C	2.80E-13	0.33	1.20E-09	−2.92	6.07	BLUEs
PLH	BLINK	**RAC875_c17628_867**	2A	90764012	G/A	1.50E-08	0.44	1.90E-05	2.22	5.57	BLUEs
PLH	BLINK	**tplb0057m23_716**	5A	475467251	A/G	1.80E-12	0.41	7.74E-09	2.80	4.44	BLUEs
PLH	BLINK	wsnp_Ex_c12063_19310114	1B	52901656	G/A	2.80E-11	0.29	7.33E-08	3.11	5.07	BLUEs
PLH	BLINK	wsnp_Ku_c21275_31007309	5A	565550079	C/A	2.40E-11	0.45	7.33E-08	2.73	4.08	BLUEs
PLH	FarmCPU	AX-94476767	5B	20833298	A/C	7.20E-08	0.25	1.54E-04	−2.77	8.5	BLUEs
PLH	FarmCPU	**AX-94506854**	1B	613423513	T/G	3.70E-11	0.05	2.38E-07	−6.40	1.64	BLUEs
PLH	FarmCPU	**BobWhite_c26893_161**	3A	502268559	C/T	1.30E-10	0.49	4.30E-07	3.11	0	BLUEs
PLH	FarmCPU	Ex_c40210_281	4A	56501947	C/T	9.70E-09	0.34	2.50E-05	2.68	23.84	BLUEs
PLH	FarmCPU	RFL_Contig1896_1236	3A	25000378	C/T	1.09E-06	0.36	1.76E-03	−2.20	3.16	BLUEs
PLH	FarmCPU	**tplb0057m23_716**	5A	475467251	A/G	3.00E-17	0.41	3.87E-13	4.54	0	BLUEs
PLH	FarmCPU	**wsnp_Ra_rep_c69692_67234463**	3A	615275885	C/T	2.20E-07	0.40	4.07E-04	−2.69	6.29	BLUEs
TKW	BLINK	RAC875_c16993_444	2B	786105655	C/T	2.55E-07	0.41	1.65E-03	1.95	13.04	Merch
TKW	BLINK	**RFL_Contig1793_315**	2A	570189823	C/T	2.76E-07	0.12	3.58E-03	1.29	24.06	Merch
TKW	BLINK	wsnp_Ex_c2054_3852564	7D	61227585	C/T	1.95E-06	0.26	1.26E-02	0.96	10.38	Merch
TKW	BLINK	wsnp_Ex_c37749_45436366	6D	178681715	T/G	3.50E-08	0.24	4.58E-04	−4.03	21.36	Merch
TKW	FarmCPU	AX-94992693	6D	23770609	T/C	2.90E-12	0.20	3.80E-08	−1.69	0.75	Merch
TKW	FarmCPU	**RFL_Contig1793_315**	2A	570189823	C/T	1.92E-06	0.12	1.24E-02	1.13	0	Merch

Trait-wise dissection of the significant MTAs revealed key associations for DHE, DMA, PLH, GY and TKW, including both pleiotropic loci and environment-specific signals. For DHE, SNP AX-94520044 on chromosome 7A exhibited the strongest association in both the BLINK and FarmCPU models, accounting for 27.11% and 14.29% of the phenotypic variance (PVE), respectively. This locus was concurrently associated with DMA, suggesting a pleiotropic role (14.29% PVE). Additional loci associated with DHE included tplb0057m23_716 (5A), Tdurum_contig17697_675 (7B), and Tdurum_contig58312_119 (7A).

For DMA, SNP AX-94506854 on chromosome 1B demonstrated the most significant association, explaining 43.47% of the variance, and was likewise associated with PLH (35.31% PVE). Markers on chromosomes 5A and 7A contributed to additional variance. On 5A, tplb0057m23_716 accounted for 3.19% of the PVE and was jointly associated with DHE (10.76% PVE) and PLH (4.44% PVE). On 7A, BS00020236_51 contributed 5.65% of the variance for DMA. Additionally, wsnp_BF474615A_Ta_1_1 on chromosome 4A and Excalibur_c21739_688 on chromosome 7B were significantly associated with DMA, with PVE values of 12.90% and 3.07%, respectively.

For GY, based on BLUEs, AX-95216538 on chromosome 2D was the only marker significantly associated across all environments, explaining 20.77% of the phenotypic variance. Environment-specific associations were also identified: wsnp_Ex_c11976_19193550 on chromosome 1B was significantly associated with GY in the Sidi El Aidi (SEA) environment (34.53% PVE), while tplb0049b24_1152 on chromosome 2D was significant in Merchouch, explaining 28.88% of the variance.

For TKW, no significant associations were detected using BLUEs or within the Terbol dataset. However, in the Merchouch dataset, several SNPs were identified as significant using both the BLINK and FarmCPU models. Notably, RAC875_c16993_444 on chromosome 2B and RFL_Contig1793_315 on chromosome 2A were associated with TKW, explaining 13.04% and 24.06% of the variance, respectively. Similarly, wsnp_Ex_c37749_45436366 on chromosome 6D was associated with 21.36% of the variance.

### Putative candidate genes associated with MTAs

A total of 37 significant MTAs were identified through GWAS for key agronomic traits under heat and drought stress. Among these, 28 were located within or near (<5 kb) annotated genes (S2 Table), spanning 12 chromosomes. Of the 28 gene-associated MTAs, 14 were intragenic, and 14 were located within 5 kb upstream or downstream of annotated gene models. For example, AX-94393508 on chromosome 2B is located within the gene *TraesCS2B02G379400* (Protein kinase) and lies near *TraesCS2B02G379300* (Glycosyltransferase). Similarly, AX-94476767 (5B), was found within *TraesCS5B02G022300* (CN hydrolase) and in close proximity to *TraesCS5B02G022400* (FAF domain). On chromosome 2A, RFL_Contig1793_315 overlapped with *TraesCS2A02G336500* (anthranilate synthase) and was located 2,988 bp upstream of *TraesCS2A02G336600* (Vacuolar iron transporter). Additional SNPs such as Ex_c40210_281 (4A), wsnp_Ra_rep_c69692_67234463 (3A), and tplb0057m23_716 (5A) were located directly within annotated gene regions. The identified genes represents diverse functional categories, including kinases, transferases, transcription factors, hydrolases, and membrane transporters, many of which are known to play roles in stress perception, signal transduction, and metabolic regulation. For instance, *TraesCS3B02G133000* encodes a DUF295 domain-containing protein involved in various biological processes, such as protein degradation, signal transduction, and stress responses, while *TraesCS6D02G348800* encodes a serine/threonine kinase, a class often implicated in stress-responsive signaling cascades. The complete list of SNPs, gene coordinates, distances, predicted protein domains, molecular functions, and associated biological processes is provided in S2 Table.

## Discussion

Enhancing wheat production requires a comprehensive analysis of its agronomic traits, as their phenotypic expression is strongly influenced by environmental variability and genotype-by-environment (G × E) interactions. Breeders have increasingly focused on discovering and deploying novel genes and QTLs associated with stress tolerance and productivity traits [34]. In this context, GWAS have become a powerful tool to dissect complex traits in wheat and to identify SNP markers associated with agronomically important characteristics such as DHE, DMA, PLH, TKW and GY. While several QTLs and MTAs have previously been reported for these traits [18,35–37], further investigations using genetically diverse material and multi-environment testing are essential to uncover novel, stable, and pleiotropic loci suitable for breeding applications. In this study, genome-wide SNP genotyping and phenotypic evaluation across seven diverse environments were used to investigate 191 elite ICARDA-developed spring wheat genotypes. This approach allowed for the identification of significant MTAs and candidate genes with potential application in improving wheat resilience to drought and heat stress in the WANA region.

### Phenotypic variation across environments

Phenotypic variance across the tested environments confirmed the influence of both environmental and genetic factors on trait expression, as reported by Ed-Daoudy et al. [18]. High CV values for traits such as GY in Wad Madani (15.37%) and Merchouch (18.74%), and TKW in Merchouch (12.98%), reflect strong environmental effects under heat and drought stress, respectively. In contrast, irrigated environments like Terbol and AREC exhibited more stable performances and higher mean yields, corroborating the observation that genotypic behavior tends to stabilize under optimal growing conditions [18]. Furthermore, these findings align with other studies indicating that wheat genotypes with reduced DHE and DMA can better adapt to drought-prone regions by escaping terminal drought stress [38–41]. Heritability analysis (*H*^*2*^) revealed that TKW, PLH and DHE exhibited high to moderate values across environments (*H*^*2*^_*across*_ = 0.761 and 0.61, respectively), suggesting strong genetic control and high potential for response to selection. These traits are, therefore, prime candidates for marker-assisted breeding under both stress and optimal conditions. Conversely, GY exhibited consistently low heritability, particularly at drought- and heat-stress-prone locations such as Sidi El Aidi (0.03) and Wad Madani (0.07), indicating a greater influence of environmental variability, which is often beyond the breeder’s control. This highlights the limitations of direct selection for GY under stress conditions and underscores the need for indirect selection strategies based on more stable traits [42].

The correlation analysis revealed strong positive associations among DHE, DMA, and PLH, indicating shared genetic regulation. In contrast, GY showed negative correlations with these traits and with TKW, which is generally considered to contribute positively to yield. However, under terminal stress conditions, particularly heat and drought, the grain-filling period is often significantly shortened. This limits the duration available for assimilate accumulation in grains, leading to trade-offs between grain number and grain weight. In such cases, plants may allocate more resources to fewer surviving grains, resulting in increased TKW but reduced total grain yield. This phenomenon has been reported previously by Lopes et al. [43] in Sudan, where TKW was negatively associated with grain yield under heat and drought stress.

These findings highlight that while TKW showed consistently high heritability across environments (*H²*_*within*_ = 0.72–0.90), and thus offers promise for selection under both optimal and stress conditions, its negative correlation with GY under heat and drought stress emphasizes the limitations of using TKW as a sole selection criterion. Therefore, selection in drought- and heat-prone environments should follow a multi-trait strategy that integrates early maturity (DHE/DMA) for drought escape, spike fertility as an indicator of sink strength, semi-dwarf plant stature to enhance assimilate partitioning toward the spike, thereby improving both spike fertility and grain yield under stress, and TKW for stable grain filling. Such an integrated approach is more effective in ensuring genetic gain for yield under variable climatic conditions.

### MTAs, putative genes and comparison with previous studies

The GWAS conducted using BLINK and FarmCPU models identified 37 significant MTAs across five key agronomic traits: DHE, DMA, PLH, TKW, and GY. These MTAs were consistently detected across seven contrasting environments, underscoring their potential utility for marker-assisted selection (MAS) in diverse agro-ecological zones, particularly those affected by drought and heat stress. Several SNPs were associated with more than one trait, suggesting pleiotropic effects and enhancing their utility in multi-trait improvement strategies.

Among these, the SNP tplb0057m23_716 on chromosome 5A was notably associated with DHE, DMA, and PLH across multiple models and environments. This SNP resides within *TraesCS5A02G261900*, which encodes eukaryotic translation initiation factor 3 subunit E (*eIF3e*), a gene previously implicated in the regulation of flowering time, plant stature, and reproductive development under abiotic stress [44,45]. The stable expression of this SNP across environments and its involvement in developmental regulation make it a prime candidate for selecting early-maturing, semi-dwarf phenotypes, particularly beneficial for stress avoidance in drought-prone regions. Furthermore, its known association with resistance to *Septoria tritici blotch* (STB) via the QTL *QStb.teagasc-5A.1* adds to its breeding relevance [46].

In relation to maturity traits, Excalibur_c21739_688 (7B) and wsnp_BF474615A_Ta_1_1 (4A) were associated to DMA and mapped near genes encoding glycosyltransferases and to QTL *QGfd.hs-4A*, respectively. These loci are functionally associated with protein N-glycosylation [41] and grain-filling duration under heat stress [47], processes known to enhance reproductive success and yield stability under terminal drought. Similarly, PLH-associated SNPs were co-localized with genes involved in hormone signaling and stress responses. For example, AX-94393508 on 2B was located within a gene encoding a protein kinase and near UDP-glucosyltransferase (*TraesCS2B02G379300*), both involved in hormonal and structural adaptations under stress [48–50]. This SNP was also previously associated with *Fusarium* head blight (FHB) resistance [51]. Likewise, Kukri_c46276_63, which is associated with both DMA and PLH, is located within the gene *TraesCS5B02G017100*, encoding a phosphatidylinositol-specific phospholipase C X domain-containing protein (*PI-PLC*). In wheat, *PI-PLC* genes have been studied for their roles in abiotic stress responses, particularly for enhancing drought and salt tolerance [52]. Notably, the same SNP has also been reported in association with the QTL *Qfhb-5B-1*, which is linked to the FHB index [53], suggesting a dual role in mediating both abiotic and biotic stress tolerance mechanisms.

Another SNP, AX-94476767 (5B), mapped to *TraesCS5B02G022300*, encoding a polyamine biosynthesis gene (*TaNPLP1–1*) implicated in drought defense through modulation of oxidative stress responses [54]. Moreover, Ex_c40210_281 (4A), associated with PLH, overlapped with *TraesCS4A02G060200*, a bHLH transcription factor (*CIB5*) involved in photoperiod and flowering regulation [55,56], consistent with observed trait correlations. Another SNP, AX-94775879, located on chromosome 2A, was associated to a putative receptor-like protein kinase [57], potentially implicated in controlling plant height in wheat [58].

For grain yield, tplb0049b24_1152 (2D) was located within the gene *TraesCS2D02G583000*, which encodes a peroxidase involved in reactive oxygen species (ROS) detoxification [59,60], and has previously been associated with Na⁺ accumulation and photosynthetic stability under osmotic stress [61,62]. Likewise, wsnp_Ex_c11976_19193550 (1B) was located within *TraesCS1B02G100600*, which encodes a *MYB* transcription factor. The *MYB* transcription factor family are well-documented regulators of plant responses to various abiotic stresses, including drought, temperature extremes, and salinity [63]. Specifically, the expression of *TraesCS1B02G100600* (*TaMYB75*) is significantly upregulated under cold stress conditions below −5 °C [64]. Additionally, the same gene has been identified as differentially expressed in response to silicon treatment in wheat, indicating a potential role in stress signaling and adaptive physiological responses [65]. TKW-related MTAs were particularly prominent under severe drought conditions in Merchouch. For example, wsnp_Ex_c37749_45436366 (6D) was associated with QTLs governing grain size traits [66], and wsnp_Ex_c2054_3852564 (7D) overlapped with *TraesCS7D02G102000*, encoding 3-ketoacyl-CoA synthase, a key enzyme in cuticular wax biosynthesis that reduces water loss and enhances drought tolerance [67]. Collectively, these findings highlight several high-value MTAs that are biologically meaningful. The co-localization of significant SNPs with genes involved in stress adaptation pathways, such as polyamine metabolism, ROS detoxification, wax biosynthesis, and hormonal regulation, supports their potential as functional markers in MAS. SNPs such as tplb0057m23_716, tplb0049b24_1152, wsnp_BF474615A_Ta_1_1, and AX-94393508 exemplify loci that not only influence multiple traits but are also tightly linked (<1 kb) to genes underpinning key adaptive and developmental processes.

## Conclusion

This study demonstrates the value of dissecting the genetic basis of key agronomic traits to enhance wheat resilience to climate stress in the WANA region. By analyzing 191 spring bread wheat genotypes across seven environments, we identified 37 significant MTAs, including five pleiotropic loci influencing phenology and plant architecture (DHE, DMA, PLH). Several SNPs co-localized with biologically relevant candidate genes, such as *TraesCS5A02G261900* (*eIF3e*), *TraesCS7D02G102000* (cuticular wax biosynthesis), *TraesCS1B02G100600* (MYB transcription factor), and *TraesCS2D02G583000* (peroxidase activity), reinforcing their roles in stress adaptation. The BLINK model proved more effective than FarmCPU in detecting significant associations, particularly under low MAF and complex LD structure. Strong phenotypic correlations and high heritability of traits such as DHE, PLH and TKW suggest these can serve as robust indirect selection criteria in environments where GY is highly variable and environment-dependent. Ultimately, integrating GWAS findings with candidate gene annotation and trait heritability provides a valuable roadmap for breeders. The identified SNPs represents excellent candidates for conversion into KASP markers, facilitating marker-assisted and genomic selection strategies to develop climate-resilient, high-yielding wheat cultivars tailored to the diverse agro-ecological conditions of the WANA region.

## Supporting information

S1 Table Adjusted phenotypic means (BLUEs) for five different agronomic traits in a panel of 191 spring bread wheat genotypes used for association mapping. (DOCX)

S1 Fig Distribution and density of 12,954 high-quality SNPs across the 21 bread wheat chromosomes. A) shows the number of SNPs per chromosome, while B) shows SNP density within 1 Mb windows across each chromosome, as used in the GWAS of the present study.
(TIF)

S2 Table Putative candidate genes located within genomic regions associated with five agronomic traits in wheat. (DOCX)
