# Supplementary material for: Identification of novel marker-trait associations for agronomic traits in bread wheat under WANA environments through GWAS
Source: PLoS One. 2025 Aug 8;20(8):e0329681. doi: 10.1371/journal.pone.0329681 (PMC12334036; doi:10.1371/journal.pone.0329681)
Supplement: S1 Table — (DOCX) [file pone.0329681.s001.docx]

**Supplementary Information**

**S1 Table**. **Adjusted phenotypic means (BLUEs) for five agronomic traits in a panel of 191 spring bread wheat genotypes used for association mapping.**

| **Sl N°** | **Name/Pedigree** | **DHE** | **DMA** | **PLH** | **TKW** | **GY** |
| --- | --- | --- | --- | --- | --- | --- |
| **G1** | Terbol | 122.647 | 167.974 | 123.816 | 37.603 | 6.274 |
| **G2** | Atlas | 120.992 | 167.379 | 109.612 | 40.384 | 4.895 |
| **G3** | Tesfa | 118.215 | 170.236 | 128.548 | 39.245 | 6.000 |
| **G4** | 22SAWSN - 142/ETBW 4921/6/HPO/TAN//VEE/3/2*PGO/4/MILAN/5/SSERI1 | 114.483 | 165.582 | 114.473 | 48.003 | 7.112 |
| **G5** | 22SAWSN - 142/ETBW 4921/6/HPO/TAN//VEE/3/2*PGO/4/MILAN/5/SSERI1 | 112.761 | 165.386 | 112.845 | 42.044 | 6.052 |
| **G6** | ABIER-2/QADANFER-4 | 106.038 | 149.312 | 86.480 | 35.866 | 7.168 |
| **G7** | ABIER-2/QADANFER-4 | 104.202 | 146.378 | 78.023 | 32.976 | 7.395 |
| **G8** | ABIER-2/QADANFER-4 | 109.008 | 148.993 | 78.901 | 28.957 | 6.784 |
| **G9** | ABU-REYAA-1/LEITH-1 | 108.339 | 148.860 | 60.471 | 36.812 | 5.965 |
| **G10** | ABU-REYAA-1/LEITH-1 | 110.395 | 150.168 | 63.172 | 38.647 | 5.858 |
| **G11** | ABUZIG-10//2*PFAU/MILAN | 116.896 | 159.181 | 109.263 | 35.043 | 6.711 |
| **G12** | ABUZIG-10//2*PFAU/MILAN | 117.614 | 159.043 | 112.755 | 35.257 | 6.894 |
| **G13** | ALMAZ-11/3/PASTOR/FLORKWA-1//PASTOR | 108.258 | 160.421 | 82.410 | 36.440 | 6.216 |
| **G14** | ALMAZ-15/SOMAMA-3 | 114.838 | 164.453 | 105.562 | 32.064 | 6.767 |
| **G15** | ALMAZ-19/ETBW 4919/3/NING MAI 9558//CHIL/CHUM18 | 109.745 | 154.601 | 86.636 | 35.047 | 6.822 |
| **G16** | ALMAZ-21/TAZA-1/3/NING MAI 9558//CHIL/CHUM18 | 105.774 | 147.819 | 80.655 | 35.043 | 6.839 |
| **G17** | ANBER-6//WORRAKATTA/PASTOR | 110.445 | 155.267 | 85.812 | 33.168 | 6.342 |
| **G18** | ASEEL-1//MILAN/PASTOR/3/SHAMISS-3 | 101.976 | 149.326 | 74.247 | 35.198 | 7.444 |
| **G19** | ASEEL-1//MILAN/PASTOR/3/SHAMISS-3 | 106.160 | 156.330 | 82.444 | 35.909 | 7.858 |
| **G20** | ASEEL-1//SHUHA-4/CHAM-8 | 116.855 | 159.974 | 91.245 | 32.562 | 6.606 |
| **G21** | ATTILA*2/AMAD//ENKOY/3/PFAU/MILAN | 125.759 | 174.762 | 138.433 | 44.395 | 5.918 |
| **G22** | ATTILA*2/AMAD//ENKOY/3/PFAU/MILAN | 120.860 | 171.618 | 131.062 | 38.860 | 6.893 |
| **G23** | ATTILA/3*BCN//ETBW 4919/4/BOW/PRL//BUC/3/WH576 | 117.393 | 162.986 | 114.206 | 32.439 | 6.587 |
| **G24** | ATTILA/3*BCN//ETBW 4919/4/BOW/PRL//BUC/3/WH576 | 116.963 | 160.073 | 103.764 | 27.436 | 6.456 |
| **G25** | ATTILA/3*BCN//MILAN/DUCULA | 120.566 | 171.334 | 132.140 | 32.313 | 6.560 |
| **G26** | ATTILA/3*BCN//MILAN/DUCULA | 117.677 | 165.898 | 125.433 | 33.830 | 7.408 |
| **G27** | ATTILA/3/URES/PRL//BAV92/4/WBLL1/5/ATTILA-3//NESMA*2/14-2/3/NS732/HER//KAUZ'S' | 114.609 | 156.623 | 89.976 | 32.698 | 6.075 |
| **G28** | ATTILA-1/NS732/HER//PARUS/PASTOR/3/TEMPORALERA M 87*2/KONK | 115.993 | 168.179 | 120.463 | 38.103 | 7.258 |
| **G29** | ATTILA-1/NS732/HER//PARUS/PASTOR/3/TEMPORALERA M 87*2/KONK | 115.100 | 165.879 | 116.362 | 41.692 | 6.613 |
| **G30** | AZD-2//PFAU/MILAN | 116.694 | 159.954 | 105.464 | 36.789 | 6.848 |
| **G31** | BAASHA-14//PFAU/MILAN | 120.980 | 162.871 | 102.639 | 38.251 | 6.144 |
| **G32** | BAASHA-14//PFAU/MILAN/3/SHAMIEKH-2 | 121.029 | 169.038 | 118.443 | 36.588 | 5.428 |
| **G33** | BABAX/LR42//BABAX*2/3/KUKUNA/4/ATTILA*2/CROW | 116.270 | 168.467 | 127.302 | 41.389 | 5.991 |
| **G34** | BACANORA 86//TAST/TORIA/3/PFAU/MILAN | 120.603 | 164.146 | 124.897 | 38.363 | 6.811 |
| **G35** | BACANORA T 88/RUTH-2 | 116.154 | 165.112 | 110.076 | 39.341 | 6.108 |
| **G36** | BACANORA T 88/RUTH-2 | 122.267 | 170.216 | 105.179 | 37.354 | 5.332 |
| **G37** | BAOBAB-1//MILAN/PASTOR | 104.028 | 155.325 | 80.857 | 38.255 | 7.109 |
| **G38** | BOW/PRL//BUC/3/WH576/4/ETBW 4919 | 112.652 | 152.628 | 85.895 | 32.915 | 7.763 |
| **G39** | CHAM-4//NS732/HER/3/ETBW 4920/5/CHEN/AEGILOPS SQUARROSA (TAUS)//BCN/3/VEE#7/BOW/4/PASTOR | 108.392 | 157.141 | 81.565 | 35.070 | 6.756 |
| **G40** | CHAM-6/SHUHA-14//MOONTASIR-2 | 115.732 | 161.904 | 112.829 | 35.682 | 7.666 |
| **G41** | CHAM-6/SHUHA-14/5/KAUZ/3/MYNA/VUL//BUC/FLK/4/MILAN | 115.571 | 161.534 | 112.587 | 37.216 | 7.123 |
| **G42** | CHAM-6/SHUHA-14/5/KAUZ/3/MYNA/VUL//BUC/FLK/4/MILAN | 116.022 | 162.345 | 113.076 | 38.423 | 7.151 |
| **G43** | CHAM-6/SHUHA-14/5/KAUZ/3/MYNA/VUL//BUC/FLK/4/MILAN | 115.938 | 162.293 | 119.729 | 34.422 | 6.466 |
| **G44** | CHAM-8/FLAG-3//MILAN/PASTOR | 104.594 | 145.246 | 64.844 | 37.838 | 6.730 |
| **G45** | CHAM-8/RUTH-3 | 113.171 | 154.042 | 95.679 | 35.028 | 7.807 |
| **G46** | CHAM-8/RUTH-3//ZAIN-2 | 123.360 | 178.676 | 144.260 | 39.941 | 7.047 |
| **G47** | CHAMRAN//SHUHA-8/DUCULA | 107.902 | 161.258 | 96.778 | 38.730 | 7.011 |
| **G48** | CHAMRAN//SHUHA-8/DUCULA | 111.745 | 161.693 | 103.897 | 40.989 | 7.090 |
| **G49** | CHAMRAN/4/OPATA/BOW//BAU/3/OPATA/BOW/5/SAMIRA-9 | 116.298 | 164.593 | 103.756 | 37.723 | 6.157 |
| **G50** | CHAMRAN/4/OPATA/BOW//BAU/3/OPATA/BOW/5/SAMIRA-9 | 115.869 | 171.503 | 145.786 | 42.400 | 6.471 |
| **G51** | CMH82A.1294/2*KAUZ//MUNIA/CHTO/3/MILAN/4/P1.861/RDWG | 110.876 | 158.231 | 93.837 | 36.698 | 6.970 |
| **G52** | CMH82A.1294/2*KAUZ//MUNIA/CHTO/3/MILAN/4/P1.861/RDWG | 112.414 | 156.660 | 94.576 | 35.959 | 6.228 |
| **G53** | CROC-1/AE.SQUARROSA (205)//MILAN/KAUZ/3/MILAN/PASTOR | 108.968 | 160.620 | 90.876 | 36.386 | 7.145 |
| **G54** | CROC-1/AE.SQUARROSA (205)//MILAN/KAUZ/3/MILAN/PASTOR | 108.001 | 160.214 | 89.283 | 39.067 | 6.692 |
| **G55** | DAJAJ-5/4/CHEN/AEGILOPS SQUARROSA (TAUS)//BCN/3/KAUZ/5/WBLL1*2/KIRITATI | 118.789 | 164.808 | 112.081 | 39.687 | 7.846 |
| **G56** | DAJAJ-5/4/CHEN/AEGILOPS SQUARROSA (TAUS)//BCN/3/KAUZ/5/WBLL1*2/KIRITATI | 118.072 | 168.842 | 117.032 | 41.388 | 7.210 |
| **G57** | DAJAJ-5/4/CHEN/AEGILOPS SQUARROSA (TAUS)//BCN/3/KAUZ/5/WBLL1*2/KIRITATI | 117.863 | 167.714 | 120.331 | 35.144 | 7.085 |
| **G58** | DAJAJ-5/ETBW 4922//NEJMAH-9 | 115.267 | 158.559 | 103.756 | 38.671 | 5.568 |
| **G59** | DEBEIRA//MILAN/PASTOR | 105.011 | 154.265 | 75.546 | 39.223 | 7.432 |
| **G60** | DEBEIRA//SHUHA-8/DUCULA | 109.990 | 156.751 | 95.813 | 29.998 | 6.471 |
| **G61** | DEBEIRA//SHUHA-8/DUCULA/3/PASTOR/SERI//PFAU | 107.232 | 156.040 | 102.960 | 36.139 | 7.109 |
| **G62** | DEBEIRA//SHUHA-8/DUCULA/3/PASTOR/SERI//PFAU | 113.947 | 165.512 | 111.149 | 36.359 | 6.741 |
| **G63** | DEBEIRA//SHUHA-8/DUCULA/3/PASTOR/SERI//PFAU | 109.308 | 157.536 | 103.152 | 33.215 | 7.171 |
| **G64** | DEBEIRA/ETBW 4922//SKAUZ/BAV92 | 104.544 | 155.376 | 91.824 | 44.362 | 6.591 |
| **G65** | DEZ//MILAN/PASTOR/3/PASTOR/SERI//PFAU | 116.485 | 166.112 | 117.102 | 41.027 | 6.838 |
| **G66** | DURRA-2/TAZA-2 | 108.962 | 154.870 | 79.606 | 37.796 | 7.177 |
| **G67** | EALME4SA - 464/7/KEA/TAN/4/TSH/3/KAL/BB//TQFN/5/PAVON/6/SW89.3064/8/WBLL1*2/BRAMBLING | 113.564 | 166.872 | 116.669 | 43.163 | 6.737 |
| **G68** | EALME4SA -167/FLAG-1/3/PASTOR/SERI//PFAU | 109.864 | 154.842 | 78.286 | 36.220 | 6.829 |
| **G69** | FARAH-7/SHAMIEKH-4 | 112.552 | 157.238 | 91.582 | 33.330 | 7.284 |
| **G70** | FARIS-17/ETBW 4920/3/MUNIA/CHTO//MILAN | 113.026 | 159.073 | 92.125 | 38.380 | 6.521 |
| **G71** | FARIS-6//PFAU/MILAN | 117.871 | 159.298 | 104.029 | 39.464 | 6.785 |
| **G72** | FAYEQ-2/3/NESMA*2/14-2//2*SAFI-3 | 116.071 | 169.911 | 122.452 | 39.784 | 6.956 |
| **G73** | FAYEQ-2/3/NESMA*2/14-2//2*SAFI-3 | 114.480 | 166.525 | 106.720 | 39.683 | 6.506 |
| **G74** | FILIN/3/CROC-1/AE.SQUARROSA (205)//KAUZ/4/FILIN/5/VEE/MJI//2*TUI/3/PASTOR/6/ASEEL-4 | 106.903 | 156.043 | 92.754 | 41.343 | 6.194 |
| **G75** | FILIN/3/CROC-1/AE.SQUARROSA (205)//KAUZ/4/FILIN/5/VEE/MJI//2*TUI/3/PASTOR/6/SHUHA-4/CHAM-12 | 110.850 | 155.998 | 90.857 | 35.536 | 7.095 |
| **G76** | FLORKWA-2/85 Z 1284//ETBW 4920/3/LOULOU-18 | 113.341 | 158.216 | 88.446 | 36.408 | 6.149 |
| **G77** | FLORKWA-2/BAASHA-25//BUSHRAA-5 | 117.409 | 161.874 | 97.868 | 36.669 | 6.451 |
| **G78** | FOW-1/SHUHA-8//ETBW 4921/3/MILAN/PASTOR | 103.936 | 154.696 | 80.683 | 38.192 | 7.787 |
| **G79** | GALVEZ/WEAVER/3/VORONA/CNO79//KAUZ/4/MILAN//PSN/BOW | 112.984 | 158.360 | 99.641 | 38.578 | 6.526 |
| **G80** | GEMMEIZA-10/SHAMISS-3 | 109.797 | 150.255 | 91.596 | 38.096 | 7.522 |
| **G81** | GOUMRIA-3//PFAU/MILAN | 121.989 | 164.833 | 124.991 | 37.604 | 6.718 |
| **G82** | GOUMRIA-3//PFAU/MILAN/3/QADANFER-4 | 121.630 | 168.023 | 132.797 | 37.527 | 6.685 |
| **G83** | HADIAH-14/QAMAR-1 | 105.641 | 154.701 | 79.638 | 29.978 | 7.465 |
| **G84** | HAMAM-4/FAISAL-1 | 104.482 | 153.334 | 84.977 | 38.386 | 7.312 |
| **G85** | HUBARA-1//ACHTAR/INRA 1764 | 111.497 | 159.005 | 94.211 | 33.613 | 6.656 |
| **G86** | HUBARA-1/5/KAUZ/3/MYNA/VUL//BUC/FLK/4/MILAN | 117.940 | 165.169 | 109.275 | 38.085 | 6.208 |
| **G87** | HUBARA-1/5/KAUZ/3/MYNA/VUL//BUC/FLK/4/MILAN | 107.671 | 152.009 | 63.255 | 32.295 | 6.224 |
| **G88** | HUBARA-1/ETBW 4921 | 120.291 | 165.641 | 101.805 | 40.958 | 5.255 |
| **G89** | HUBARA-1/ETBW 4921 | 117.981 | 161.416 | 94.705 | 43.282 | 6.269 |
| **G90** | HUBARA-13//ACHTAR/INRA 1764 | 113.764 | 159.754 | 87.530 | 36.356 | 6.562 |
| **G91** | HUBARA-13//ACHTAR/INRA 1764 | 112.812 | 158.940 | 87.558 | 33.512 | 6.924 |
| **G92** | HUBARA-13/ETBW 4919//KINGBIRD | 114.727 | 160.635 | 112.852 | 38.099 | 6.076 |
| **G93** | HUBARA-16//RDWG/MILAN | 118.391 | 161.861 | 114.215 | 37.857 | 6.554 |
| **G94** | HUBARA-5//PFAU/MILAN | 121.141 | 164.444 | 105.193 | 38.782 | 6.111 |
| **G95** | HUBARA-5/3/NESMA*2/261-9//FIRETAIL | 118.944 | 165.874 | 106.014 | 38.592 | 6.256 |
| **G96** | INQALAB91*2/TUKURU//MILAN/PASTOR | 106.420 | 153.885 | 75.530 | 37.947 | 6.971 |
| **G97** | JAWAHIR-10//SHUHA-8/DUCULA | 106.994 | 151.140 | 83.164 | 34.134 | 7.321 |
| **G98** | JAWAHIR-10//SHUHA-8/DUCULA | 106.420 | 153.885 | 75.530 | 32.731 | 7.326 |
| **G99** | JAWAHIR-10/ETBW 4922 | 106.420 | 153.885 | 75.530 | 37.947 | 6.256 |
| **G100** | JAWAHIR-2//MILAN/DUCULA | 109.473 | 150.963 | 80.485 | 35.679 | 6.379 |
| **G101** | JAWAHIR-9/ETBW 4920 | 103.946 | 143.892 | 67.051 | 35.454 | 8.072 |
| **G102** | JAWAHIR-9/QAMAR-1 | 111.079 | 156.191 | 101.638 | 34.999 | 6.929 |
| **G103** | KA/NAC//SERI/RAYON/3/GOUMRIA-14 | 119.157 | 171.622 | 132.823 | 40.918 | 5.844 |
| **G104** | KATILA-11//SHUHA-8/DUCULA | 112.254 | 161.295 | 107.955 | 37.193 | 6.926 |
| **G105** | KATILA-13//PFAU/MILAN | 120.584 | 163.214 | 111.175 | 32.772 | 6.236 |
| **G106** | KATILA-9//HXL8088/DUCULA | 105.818 | 145.160 | 72.585 | 34.909 | 6.785 |
| **G107** | KBG-01//MILAN/PASTOR | 105.416 | 154.967 | 76.140 | 30.009 | 7.029 |
| **G108** | KINGBIRD/3/NESMA*2/14-2//2*SAFI-3 | 115.690 | 170.321 | 131.938 | 40.592 | 6.196 |
| **G109** | KOUKAB-1//PFAU/MILAN | 121.241 | 165.908 | 118.795 | 35.571 | 6.032 |
| **G110** | KOUKAB-1//PFAU/MILAN/3/SOSSI-3 | 121.755 | 168.067 | 105.091 | 37.508 | 5.658 |
| **G111** | KRICHAUFF/2*PASTOR//SHUHA-8/DUCULA | 106.181 | 154.226 | 90.413 | 33.906 | 7.127 |
| **G112** | LAKTA-7/FLAG-5//NEJMAH-9 | 116.389 | 164.477 | 106.728 | 40.151 | 7.120 |
| **G113** | LFN/II58.57//PRL/3/HAHN/4/KAUZ/5/KAUZ/6/NJORO SD-4 | 116.814 | 163.485 | 114.534 | 37.817 | 6.606 |
| **G114** | MASSIRA//MILAN/PASTOR | 107.540 | 159.982 | 96.543 | 39.379 | 7.303 |
| **G115** | MILAN/PASTOR//ETBW 4919 | 103.947 | 151.487 | 71.453 | 33.991 | 7.347 |
| **G116** | MILAN/PASTOR//SHUHA-8/DUCULA/3/TEMPORALERA M 87*2/KONK | 105.572 | 155.945 | 84.795 | 36.385 | 6.780 |
| **G117** | MILAN/PASTOR/3/MUNIA/CHTO//MILAN | 116.516 | 168.953 | 118.830 | 43.178 | 6.342 |
| **G118** | MILAN/PASTOR/3/MUNIA/CHTO//MILAN | 107.720 | 150.511 | 74.364 | 34.688 | 6.923 |
| **G119** | MILAN/SHA7/3/THB/CEP7780//SHA4/LIRA/4/SHA4/CHIL/5/AGUILAL | 105.336 | 151.047 | 74.654 | 37.658 | 5.890 |
| **G120** | MILAN/SHA7/3/THB/CEP7780//SHA4/LIRA/4/SHA4/CHIL/5/AGUILAL | 112.899 | 161.045 | 90.598 | 47.649 | 5.234 |
| **G121** | MO88/MILAN//ETBW 4922/3/(4) EALME4SA - 464 | 116.120 | 160.626 | 104.473 | 38.704 | 6.719 |
| **G122** | MUNIA//CHEN/ALTAR 84/3/CHEN/AEGILOPS SQUARROSA (TAUS)//BCN/4/FLAG-2 | 109.857 | 152.181 | 72.505 | 38.279 | 7.203 |
| **G123** | MUNIA//CHEN/ALTAR 84/3/CHEN/AEGILOPS SQUARROSA (TAUS)//BCN/4/FLAG-2 | 109.145 | 154.670 | 76.720 | 40.189 | 6.896 |
| **G124** | MUNIA/ALTAR 84//MILAN/3/MILAN/PASTOR | 107.418 | 153.746 | 81.592 | 41.778 | 7.340 |
| **G125** | NEJMAH-18/QADANFER-4 | 112.468 | 162.540 | 112.155 | 38.480 | 6.722 |
| **G126** | NESSER/SERI//MOONTASIR-2/3/KAUZ'S'/FLORKWA-1 | 106.883 | 153.975 | 80.632 | 36.913 | 7.700 |
| **G127** | NING MAI 9558//CHIL/CHUM18/3/SAFI-1 | 117.361 | 161.132 | 78.153 | 40.364 | 6.363 |
| **G128** | OAX93.24.35//SOKOLL/WBLL1/4/NS732/HER//ARRIHANE/3/REGRAG-1 | 108.678 | 153.832 | 88.458 | 43.625 | 7.177 |
| **G129** | OPATA/RAYON//KAUZ/3/ACHTAR/INRA 1764 | 115.291 | 162.674 | 108.146 | 38.039 | 6.053 |
| **G130** | OPATA/RAYON//KAUZ/3/MILAN/DUCULA | 112.022 | 162.785 | 115.838 | 36.817 | 7.084 |
| **G131** | OPATA/RAYON//KAUZ/3/MILAN/DUCULA | 110.279 | 162.296 | 112.377 | 37.251 | 6.815 |
| **G132** | P1.861/RDWG//ESWYT99#18/ARRIHANE/3/PFAU/MILAN | 116.851 | 164.682 | 115.468 | 33.763 | 7.066 |
| **G133** | PASTOR//HXL7573/2*BAU/3/WBLL1/4/N-AZRAQ-3 | 109.481 | 158.484 | 101.937 | 37.541 | 5.377 |
| **G134** | PBW343*2/KUKUN//ANBER-9 | 121.373 | 173.777 | 142.403 | 42.992 | 5.631 |
| **G135** | PBW343*2/KUKUN//ANBER-9 | 119.251 | 171.346 | 134.496 | 37.900 | 6.595 |
| **G136** | PBW343/ETBW 4921//QAMAR-6 | 110.489 | 157.652 | 93.526 | 38.150 | 6.896 |
| **G137** | PBW343/ETBW 4921//QAMAR-6 | 108.371 | 158.623 | 95.114 | 35.604 | 5.868 |
| **G138** | PFAU/MILAN//FLAG-3 | 114.005 | 157.835 | 105.862 | 37.634 | 6.723 |
| **G139** | PFAU/MILAN//FLAG-3/3/NEJMAH-9 | 114.897 | 160.496 | 103.363 | 40.692 | 6.524 |
| **G140** | PFAU/MILAN//FUNG MAI 24/3/ACHTAR/INRA 1764 | 113.648 | 164.328 | 110.318 | 42.009 | 6.811 |
| **G141** | PFAU/MILAN//JAWAHIR-9 | 114.020 | 152.765 | 94.714 | 32.966 | 6.982 |
| **G142** | POTAM/ESWYT99#18/6/FILIN/3/CROC-1/AE.SQUARROSA (205)//KAUZ/4/FILIN/5/VEE/MJI//2*TUI/3/PASTOR | 110.631 | 160.079 | 95.024 | 43.488 | 6.710 |
| **G143** | PRL/SARA//TSI/VEE#5/3/HUITES/4/SALE-19 | 115.188 | 161.254 | 104.745 | 37.598 | 6.659 |
| **G144** | PVN//CAR422/ANA/5/BOW/CROW//BUC/PVN/3/YR/4/TRAP#1/6/NS732/HER//SD8036/3/SAADA | 107.447 | 157.163 | 83.826 | 33.206 | 6.637 |
| **G145** | QADANFER-4//ACHTAR/INRA 1764/3/SHAMISS-3 | 108.856 | 157.622 | 91.569 | 40.301 | 6.599 |
| **G146** | QADANFER-4/3/PSN/BOW//MILAN | 106.726 | 153.929 | 90.858 | 42.551 | 7.475 |
| **G147** | QAMAR-4/3/NESMA*2/14-2//2*SAFI-3 | 115.811 | 169.829 | 127.017 | 37.125 | 6.209 |
| **G148** | QAMAR-4/3/NESMA*2/14-2//2*SAFI-3 | 116.691 | 169.823 | 124.711 | 41.432 | 6.857 |
| **G149** | QIMMA-12/ETBW 4919/4/CHEN/AEGILOPS SQUARROSA (TAUS)//FCT/3/2*WEAVER | 109.118 | 156.778 | 81.322 | 38.620 | 5.709 |
| **G150** | RABIH-10/ETBW 4922 | 110.852 | 157.425 | 79.856 | 38.189 | 6.997 |
| **G151** | RABIH-10/ETBW 4922//KAUZ'S'/FLORKWA-1 | 111.155 | 158.836 | 87.901 | 43.498 | 5.685 |
| **G152** | RABIH-3/5/KAUZ/3/MYNA/VUL//BUC/FLK/4/MILAN | 115.249 | 156.014 | 66.210 | 37.471 | 5.788 |
| **G153** | RABIH-3/5/KAUZ/3/MYNA/VUL//BUC/FLK/4/MILAN | 112.522 | 156.320 | 69.007 | 38.628 | 6.206 |
| **G154** | RABIH-3/5/KAUZ/3/MYNA/VUL//BUC/FLK/4/MILAN | 113.250 | 154.017 | 60.109 | 35.089 | 5.831 |
| **G155** | RABIH-9/ETBW 4921//SHUHA-1/DORG-1 | 111.137 | 158.244 | 109.261 | 37.474 | 7.057 |
| **G156** | SAAMID-3/3/FERROUG-1/SAFI-1//FERROUG-2 | 115.687 | 163.552 | 99.656 | 38.642 | 6.708 |
| **G157** | SERI*3//RL6010/4*YR/3/PASTOR/4/BAV92/5/ETBW 4921/6/SAMIRA-9 | 111.182 | 157.840 | 89.612 | 37.517 | 6.463 |
| **G158** | SERI*3//RL6010/4*YR/3/PASTOR/4/BAV92/5/MILAN/PASTOR/7/CHAM-8/6/SAKER'S'/5/RBS/ANZA/3/KVZ/HYS//YMH/TOB/4/BOW'S' | 111.151 | 161.337 | 87.780 | 43.886 | 6.010 |
| **G159** | SERI.1B//KAUZ/GEN/3/AMAD/4/MILAN/PASTOR | 109.677 | 153.672 | 62.021 | 41.716 | 6.496 |
| **G160** | SERI.1B//KAUZ/HEVO/3/AMAD/4/ESWYT99#18/ARRIHANE | 116.675 | 168.132 | 114.999 | 35.995 | 6.299 |
| **G161** | SERI.1B//KAUZ/HEVO/3/AMAD/4/MILAN/PASTOR | 110.222 | 159.625 | 83.262 | 39.440 | 6.037 |
| **G162** | SERI.1B//KAUZ/HEVO/3/AMAD/4/MILAN/PASTOR | 111.840 | 160.686 | 107.015 | 35.231 | 6.289 |
| **G163** | SERI.1B//KAUZ/HEVO/3/AMAD/4/MILAN/PASTOR | 110.685 | 159.386 | 91.311 | 36.715 | 6.527 |
| **G164** | SHAMIEKH-4/FLAG-8 | 112.855 | 160.337 | 90.631 | 40.964 | 6.545 |
| **G165** | SUDAN#3/SHUHA-6//FLAG-5/3/PFAU/MILAN | 120.279 | 160.965 | 108.488 | 34.467 | 6.097 |
| **G166** | SUDAN#3/SHUHA-6/4/BOW/PRL//BUC/3/WH576 | 112.205 | 160.597 | 109.238 | 31.741 | 6.235 |
| **G167** | T.TAU.83.2.36/FRAME/3/OPATA/RAYON//KAUZ | 114.035 | 164.799 | 114.962 | 42.290 | 6.644 |
| **G168** | TC870344/GUI//TEMPORALERA M 87/AGR/3/2*WBLL1/4/ABIER-2 | 115.947 | 166.074 | 117.087 | 44.554 | 7.413 |
| **G169** | TC870344/GUI//TEMPORALERA M 87/AGR/3/2*WBLL1/4/ABIER-2 | 116.577 | 164.601 | 120.503 | 39.223 | 6.792 |
| **G170** | TEMPORALERA M 87*2/KONK//FAYEQ-1 | 115.787 | 166.713 | 118.953 | 34.637 | 6.564 |
| **G171** | TEMPORALERA M 87*2/KONK//FAYEQ-1 | 116.190 | 168.224 | 119.340 | 38.963 | 7.372 |
| **G172** | TEMPORALERA M 87*2/TUKURU//FAYEQ-2 | 117.331 | 167.339 | 123.386 | 37.388 | 6.478 |
| **G173** | TEVEE'S'/SHUHA'S'//ACHTAR/INRA 1764/3/CHIL-1/SHUHA-1 | 111.295 | 157.766 | 92.430 | 38.065 | 6.738 |
| **G174** | TRAP#1/BOW//PFAU/3/MILAN/4/ETBW 4922/5/PFAU/MILAN | 114.446 | 156.251 | 94.148 | 39.023 | 7.384 |
| **G175** | V763.2312/V879.C8.11.11.11(36)//STAR/3/STAR/4/ETBW 4921/5/KAUZ'S'/FLORKWA-1 | 114.247 | 159.092 | 105.669 | 34.435 | 6.611 |
| **G176** | VEE#5/SARA//DUCULA/3/NJORO SD-7/4/PFAU/MILAN | 114.240 | 162.182 | 97.244 | 37.373 | 6.039 |
| **G177** | VEE/NAC//MILAN/PASTOR/5/HUITES/4/CS/TH.SC//3*PVN/3/MIRLO/BUC | 116.745 | 167.556 | 114.098 | 44.691 | 6.586 |
| **G178** | WATAN-6/ETBW 4919//ZAKIA-14 | 97.989 | 142.968 | 80.937 | 33.885 | 6.656 |
| **G179** | WBLL1*2/BRAMBLING/3/OPATA/RAYON//KAUZ | 110.119 | 161.450 | 128.383 | 40.293 | 6.124 |
| **G180** | WBLL4//OAX93.24.35/WBLL1/3/NESMA*2/14-2//2*SAFI-3 | 111.993 | 166.576 | 108.112 | 40.997 | 5.848 |
| **G181** | WEAVER/TSC//WEAVER/3/WEAVER/4/WAXWING/5/DURRA-8 | 115.233 | 163.170 | 103.972 | 39.528 | 6.349 |
| **G182** | WEAVER/TSC//WEAVER/3/WEAVER/4/WAXWING/5/MILAN/SHA7//POTAM*3KS811261-5 | 118.632 | 171.261 | 122.606 | 38.549 | 5.508 |
| **G183** | WHEATEAR//ACHTAR/INRA 1764 | 109.599 | 158.157 | 93.525 | 40.179 | 6.498 |
| **G184** | WHEATEAR//ACHTAR/INRA 1764 | 106.491 | 153.604 | 83.346 | 41.721 | 6.282 |
| **G185** | ZAIEM-11/PASTOR-6 | 104.176 | 154.023 | 65.828 | 36.695 | 6.971 |
| **G186** | ZAIEM-11/PASTOR-6/5/HUITES/4/CS/TH.SC//3*PVN/3/MIRLO/BUC | 109.265 | 156.580 | 82.264 | 35.404 | 5.708 |
| **G187** | ZAIEM-11/PASTOR-6/5/HUITES/4/CS/TH.SC//3*PVN/3/MIRLO/BUC | 113.282 | 160.077 | 85.369 | 36.364 | 6.068 |
| **G188** | ZAIEM-4/5/JUN//MAYA/MON/3/PGO/4/MILAN | 114.571 | 156.987 | 85.993 | 32.654 | 6.735 |
| **G189** | ZAIN-4/QADANFER-11 | 121.753 | 163.532 | 104.642 | 33.870 | 5.922 |
| **G190** | ZERBA-6/FLAG-6/3/TAM200/PASTOR//TOBA97 | 111.356 | 161.435 | 105.050 | 41.987 | 7.036 |
| **G191** | ZOOMOROUD-5/ETBW 4920/7/BACANORA 86/6/SN64/HN4//REX/3/EDCH/MEX/4/SLS'S'/5/BOW'S' | 113.222 | 159.205 | 108.124 | 35.714 | 6.440 |
