# Supplementary figures and images for: Identification of novel marker-trait associations for agronomic traits in bread wheat under WANA environments through GWAS
Source: PLoS One. 2025 Aug 8;20(8):e0329681. doi: 10.1371/journal.pone.0329681 (PMC12334036; doi:10.1371/journal.pone.0329681)

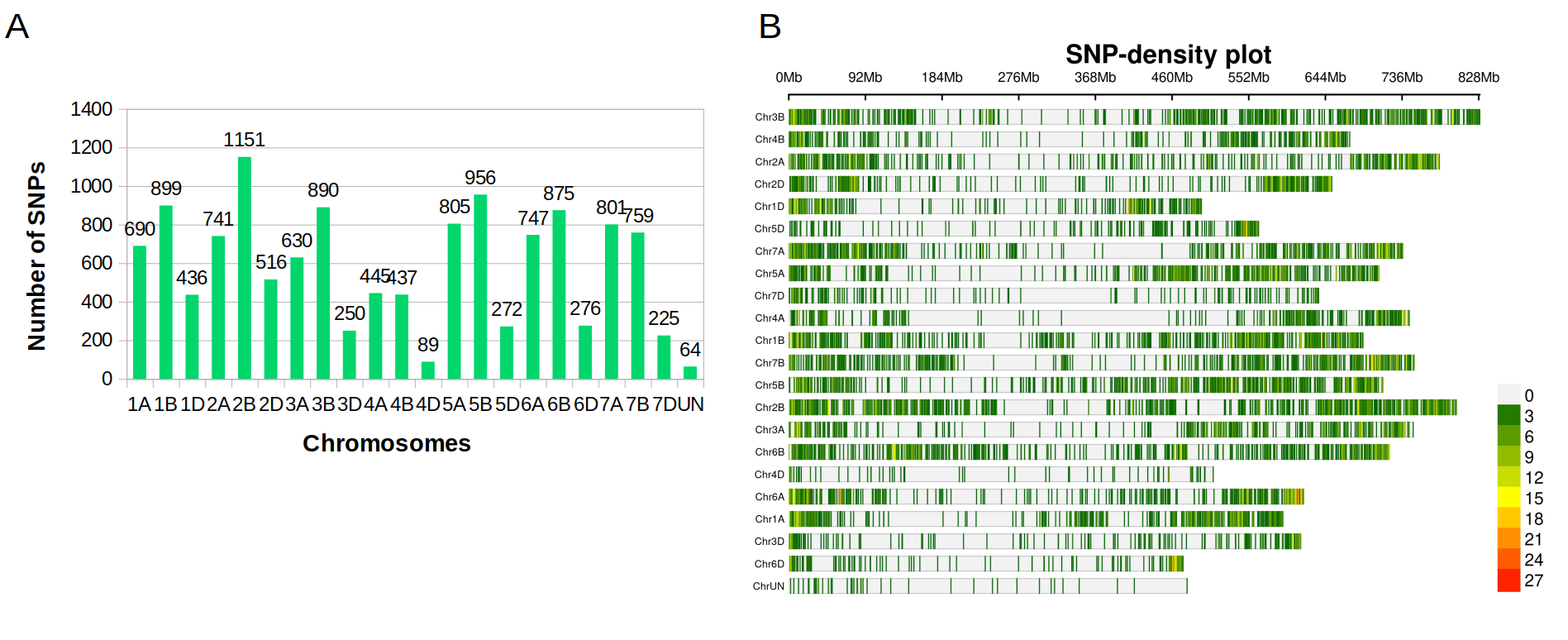

Supplement: S1 Fig — A) shows the number of SNPs per chromosome, while B) shows SNP density within 1 Mb windows across each chromosome, as used in the GWAS of the present study. (TIF) [file pone.0329681.s002.tif]
