# Supplementary material for: Identification of novel marker-trait associations for agronomic traits in bread wheat under WANA environments through GWAS
Source: PLoS One. 2025 Aug 8;20(8):e0329681. doi: 10.1371/journal.pone.0329681 (PMC12334036; doi:10.1371/journal.pone.0329681)
Supplement: S2 Table — (DOCX) [file pone.0329681.s003.docx]

**Supplementary Information**

**S2 Table.** **Putative candidate genes located within genomic regions associated with five agronomic traits in wheat.**

| **Trait** | **SNP** | **Chr** | **SNP position (bp)** | **Alleles** | **Gene** | **Gene_Start (bp)** | **Gene_End (bp)** | **Physical distance (bp)** | **Protein** | **Molecular Function** | **Biological process** |
| --- | --- | --- | --- | --- | --- | --- | --- | --- | --- | --- | --- |
| PLH | AX-94393508 | 2B | 542759611 | A/G | *TraesCS2B02G379300* | 542756627 | 542758393 | 1218 | Glycosyltransferase | UDP-glucosyltransferase activity |  |
| PLH | AX-94393508 | 2B | 542759611 | A/G | *TraesCS2B02G379400* | 542759150 | 542763986 | 0 | Protein kinase domain-containing protein | ATP binding/ protein kinase activity/ signaling receptor activity | hormone-mediated signaling pathway/ protein phosphorylation |
| PLH | AX-94452017 | 3B | 115396333 | T/C | *TraesCS3B02G132900* | 115394897 | 115395869 | 464 | Uncharacterized protein |  |  |
| PLH | AX-94452017 | 3B | 115396333 | T/C | *TraesCS3B02G133000* | 115396160 | 115399696 | 0 | DUF295 domain-containing protein |  |  |
| PLH | AX-94476767 | 5B | 20833298 | A/C | *TraesCS5B02G022300* | 20829916 | 20833469 | 0 | CN hydrolase domain-containing protein | N-carbamoylputrescine amidase activity | putrescine biosynthetic process from arginine |
| PLH | AX-94476767 | 5B | 20833298 | A/C | *TraesCS5B02G022400* | 20833769 | 20834710 | 471 | FAF domain-containing protein |  |  |
| DMA, PLH | AX-94506854 | 1B | 613423513 | T/G | *TraesCS1B02G380900* | 613416752 | 613419585 | 3928 | AB hydrolase-1 domain-containing protein | hydrolase activity |  |
| DMA | AX-94881375 | 6D | 446614420 | A/C | *TraesCS6D02G348700* | 446610282 | 446615371 | 0 | Uncharacterized protein |  | cell surface receptor signaling pathway |
| DMA | AX-94881375 | 6D | 446614420 | A/C | *TraesCS6D02G348800* | 446619401 | 446621722 | 4981 | Protein kinase domain-containing protein | ATP binding/ protein serine/threonine kinase activity | protein phosphorylation |
| PLH | AX-95255993 | 2A | 31811192 | T/C | *TraesCS2A02G071300* | 31785280 | 31811688 | 0 | Uncharacterized protein | ADP binding | defense response/ response to other organism |
| PLH | BS00021860_51 | 5A | 704973862 | T/C | *TraesCS5A02G551500* | 704972507 | 704975752 | 0 | PGG domain-containing protein |  |  |
| PLH | BS00025191_51 | 3A | 36474852 | T/G | *TraesCS3A02G060800* | 36472283 | 36475148 | 0 | GTD-binding domain-containing protein | myosin XI tail binding |  |
| PLH | BS00025191_51 | 3A | 36474852 | T/G | *TraesCS3A02G060900* | 36479502 | 36480653 | 4650 | non-specific serine/threonine protein kinase | ATP binding/ protein serine/threonine kinase activity | protein phosphorylation |
| PLH | Ex_c40210_281 | 4A | 56501947 | C/T | *TraesCS4A02G060100* | 56496985 | 56501161 | 786 | Uncharacterized protein |  | negative regulation of photomorphogenesis/ regulation of abscisic acid-activated signaling pathway |
| PLH | Ex_c40210_281 | 4A | 56501947 | C/T | *TraesCS4A02G060200* | 56501607 | 56506002 | 0 | BHLH domain-containing protein | DNA-binding transcription factor activity/ protein dimerization activity | Transcription regulation |
| DMA | Excalibur_c21739_688 | 7B | 632491797 | A/G | *TraesCS7B02G367800* | 632493372 | 632495527 | 1575 | Dolichyl-diphosphooligosaccharide--protein glycosyltransferase subunit DAD1 |  | apoptotic process/ protein N-linked glycosylation |
| PLH | IACX2946 | 6A | 599046538 | G/A | *TraesCS6A02G378000* | 599042583 | 599046908 | 0 | non-specific serine/threonine protein kinase | ATP binding/ protein serine/threonine kinase activity | endocytosis/ protein phosphorylation/ signal transduction |
| PLH | IACX2946 | 6A | 599046538 | G/A | *TraesCS6A02G378100* | 599047360 | 599051994 | 822 | Diphthine--ammonia ligase | diphthine-ammonia ligase activity | protein histidyl modification to diphthamide |
| DMA, PLH | Kukri_c46276_63 | 5B | 16002366 | T/C | *TraesCS5B02G017100* | 16001165 | 16005166 | 0 | Phosphatidylinositol-specific phospholipase C X domain-containing protein | phosphoric diester hydrolase activity | lipid metabolic process |
| DMA | Kukri_c6460_1823 | 3B | 117575406 | C/A | *TraesCS3B02G135100* | 117574881 | 117595547 | 0 | Spatacsin C-terminal domain-containing protein |  |  |
| DMA | RAC875_c16827_292 | 5B | 50469657 | C/T | *TraesCS5B02G044900* | 50468600 | 50470918 | 0 | F-box domain-containing protein |  |  |
| TKW | RAC875_c16993_444 | 2B | 786105655 | C/T | *TraesCS2B02G604500* | 786102579 | 786105550 | 105 | Protein kinase domain-containing protein | ATP binding/ calcium ion binding/ polysaccharide binding/ protein serine/threonine kinase activity | cell surface receptor signaling pathway/ protein phosphorylation |
| TKW | RAC875_c16993_444 | 2B | 786105655 | C/T | *TraesCS2B02G604600* | 786105639 | 786106031 | 0 | Serine-threonine/tyrosine-protein kinase catalytic domain-containing protein |  | cell surface receptor signaling pathway |
| DMA, PLH | RAC875_c17628_867 | 2A | 90764012 | G/A | *TraesCS2A02G146200* | 90758888 | 90763964 | 48 | Protein kinase domain-containing protein | ATP binding/ calcium-dependent protein serine/threonine kinase activity/ calcium/calmodulin-dependent protein kinase activity/ calmodulin binding | intracellular signal transduction/ protein phosphorylation |
| DMA, PLH | RAC875_c17628_867 | 2A | 90764012 | G/A | *TraesCS2A02G146400* | 90768482 | 90769814 | 4470 | MADS-box domain-containing protein | DNA-binding transcription factor activity, RNA polymerase II-specific/ protein dimerization activity/ RNA polymerase II cis-regulatory region sequence-specific DNA binding | positive regulation of transcription by RNA polymerase II |
| TKW | RFL_Contig1793_315 | 2A | 570189823 | C/T | *TraesCS2A02G336500* | 570189214 | 570192491 | 0 | anthranilate synthase |  | glutamine metabolic process/ tryptophan biosynthetic process |
| TKW | RFL_Contig1793_315 | 2A | 570189823 | C/T | *TraesCS2A02G336600* | 570192811 | 570195203 | 2988 | Vacuolar iron transporter | iron ion transmembrane transporter activity/ manganese ion transmembrane transporter activity/ metal ion binding | intracellular manganese ion homeostasis/ intracellular sequestering of iron ion |
| PLH | RFL_Contig1896_1236 | 3A | 25000378 | C/T | *TraesCS3A02G047200* | 24996681 | 25001321 | 0 | Fe-S cluster assembly protein SufD |  | iron-sulfur cluster assembly |
| DHE | Tdurum_contig17697_675 | 7B | 699147853 | G/A | *TraesCS7B02G430700* | 699148596 | 699156419 | 743 | AP180 N-terminal homology (ANTH) domain-containing protein | 1-phosphatidylinositol binding/ clathrin heavy chain binding/ phosphatidylinositol-4,5-bisphosphate binding/ SNARE binding | clathrin coat assembly/ clathrin-dependent endocytosis/ vesicle budding from membrane |
| GY | tplb0049b24_1152 | 2D | 643436189 | C/T | *TraesCS2D02G583000* | 643435027 | 643436437 | 0 | Peroxidase | heme binding/ lactoperoxidase activity/ metal ion binding/ peroxidase activity | hydrogen peroxide catabolic process/ response to oxidative stress/ response to stress |
| DHE, DMA, PLH | tplb0057m23_716 | 5A | 475467251 | A/G | *TraesCS5A02G261900* | 475464797 | 475468063 | 0 | Eukaryotic translation initiation factor 3 subunit E | translation initiation factor activity | formation of cytoplasmic translation initiation complex/ translational initiation |
| DMA | wsnp_BF474615A_Ta_1_1 | 4A | 581869247 | T/C | *TraesCS4A02G270700* | 581867995 | 581871274 | 0 | Phytanoyl-CoA dioxygenase | phytanoyl-CoA dioxygenase activity | Cofactor Fe cation |
| GY | wsnp_Ex_c11976_19193550 | 1B | 109729520 | G/A | *TraesCS1B02G100600* | 109728173 | 109730564 | 0 | Uncharacterized protein | transcription cis-regulatory region binding | regulation of DNA-templated transcription |
| PLH | wsnp_Ex_c12063_19310114 | 1B | 52901656 | G/A | *TraesCS1B02G068000* | 52897847 | 52902749 | 0 | Uncharacterized protein |  | reciprocal meiotic recombination |
| PLH | wsnp_Ex_c12063_19310114 | 1B | 52901656 | G/A | *TraesCS1B02G068100* | 52903646 | 52909197 | 1990 | NAB domain-containing protein | actin binding |  |
| TKW | wsnp_Ex_c2054_3852564 | 7D | 61227585 | C/T | *TraesCS7D02G102000* | 61227198 | 61228935 | 0 | 3-ketoacyl-CoA synthase | acyltransferase activity, transferring groups other than amino-acyl groups | fatty acid biosynthetic process |
| PLH | wsnp_Ku_c21275_31007309 | 5A | 565550079 | C/A | *TraesCS5A02G365500* | 565538552 | 565551360 | 0 | Helicase C-terminal domain-containing protein | ATP hydrolysis activity/ ATP-dependent chromatin remodeler activity/ chromatin binding/ DNA binding/ histone binding | chromatin remodeling/ heterochromatin formation |
| TKW | wsnp_Ku_c4342_7887834 | 4A | 606365805 | C/T | *TraesCS4A02G316300* | 606361307 | 606366402 | 0 | Uncharacterized protein | hydrolase activity, acting on ester bonds |  |
| DMA, PLH | wsnp_Ra_rep_c69692_67234463 | 3A | 615275885 | C/T | *TraesCS3A02G366300* | 615274210 | 615280414 | 0 | Alpha-1,4 glucan phosphorylase | glycogen phosphorylase activity/ linear malto-oligosaccharide phosphorylase activity/ pyridoxal phosphate binding/ SHG alpha-glucan phosphorylase activity | glycogen catabolic process |
